# Functional variants identify sex-specific genes and pathways in Alzheimer’s Disease

**DOI:** 10.1038/s41467-023-38374-z

**Published:** 2023-05-13

**Authors:** Thomas Bourquard, Kwanghyuk Lee, Ismael Al-Ramahi, Minh Pham, Dillon Shapiro, Yashwanth Lagisetty, Shirin Soleimani, Samantha Mota, Kevin Wilhelm, Maryam Samieinasab, Young Won Kim, Eunna Huh, Jennifer Asmussen, Panagiotis Katsonis, Juan Botas, Olivier Lichtarge

**Affiliations:** 1 Department of Molecular and Human Genetics, Baylor College of Medicine, Houston, TX 77030 USA; 2 Jan and Dan Duncan Neurological Research Institute, Texas Children’s Hospital, Houston, TX 77030 USA; 3 Center for Alzheimer’s and Neurodegenerative Diseases, Baylor College of Medicine, Houston, TX 77030 USA; 4 Department of Biology and Pharmacology, UTHealth McGovern Medical School, Houston, TX 77030 USA; 5 Computational and Integrative Biomedical Research Center, Baylor College of Medicine, Houston, TX 77030 USA

**Keywords:** Genetics, Neuroscience

## Abstract

The incidence of Alzheimer’s Disease in females is almost double that of males. To search for sex-specific gene associations, we build a machine learning approach focused on functionally impactful coding variants. This method can detect differences between sequenced cases and controls in small cohorts. In the Alzheimer’s Disease Sequencing Project with mixed sexes, this approach identified genes enriched for immune response pathways. After sex-separation, genes become specifically enriched for stress-response pathways in male and cell-cycle pathways in female. These genes improve disease risk prediction in silico and modulate *Drosophila* neurodegeneration in vivo. Thus, a general approach for machine learning on functionally impactful variants can uncover sex-specific candidates towards diagnostic biomarkers and therapeutic targets.

## Abstract (web summary)

More females than males suffer from Alzheimer’s Disease for reasons not well understood. Here, using a novel machine learning approach focused on functionally impactful coding variants, the authors identify potential sex-specific modulators of neurodegeneration.

## Introduction

Alzheimer’s Disease (AD) is a fatal neurodegenerative illness characterized by progressive dementia. Familial early onset AD (FAD, <1% AD cases)^1^ is dominantly inherited and involves mutations in *APP* ^2–4^ or *PSEN1/2*^4–7^ genes. The more prevalent sporadic late-onset AD (LOAD, >90% of cases^1^) is a complex trait disease, which stems from genetic risk^8^ and environmental factors^9–12^ with an estimated heritability of 0.60~0.80^13,14^. Genome-wide association studies (GWAS) have found more than 30 LOAD-associated loci^15–18^ accounting for ~0.33^19^ of the heritability (mostly explained by *APOEε4*)^20,21^. Often LOAD-associated GWAS loci fall in difficult to interpret non-coding regions.

Males and females differ in AD prevalence and progression. After controlling for APOE status and age^22–24^, females suffer faster cognitive loss^25^, cerebral atrophy^26,27^, and hippocampal volume loss^27^, while males experience greater mortality^28,29^. Depression^30–33^, sleep disturbances^34^, and cardiometabolic disorders^28,35^ associated with menopause combined with longer average life expectancy may partially explain the increased likelihood of AD in female^36–38^. However, the role of genetics in sex-specific AD risk has not been systematically studied. Females who carry an *APOEε4* allele have higher cerebrospinal fluid (CSF) tau levels and higher AD risk than males^39^. Gene-by-sex interaction analyses have revealed sex-specific effects on AD risk for *ACE*^40^, *BDNF*^41^ and *RELN*^42^ and a recent family-based association study found four additional genes (*GRID1*, *RIOK3*, *MCPH1*, *ZBTB7C*) that conferred a sex-specific association to AD^43^. Beyond these targeted studies, only one GWAS has been performed where the cohort was separated by sex using CSF Aβ42 and tau as endophenotypes^44^. Most large-scale genome wide meta-analyses have focused on AD status and have not attempted sex-based separation, likely due to the loss of statistical power that halving the sample entails. It is critical to identify genetic contributors that underlie sex-differences in AD as it could lead to more accurate disease risk assessment and more tailored therapeutic approaches^45,46^. Bridging this gap will require analytical methods capable of extracting meaningful genetic information from smaller samples than those used in traditional genome-wide approaches.

**Fig. 1:**
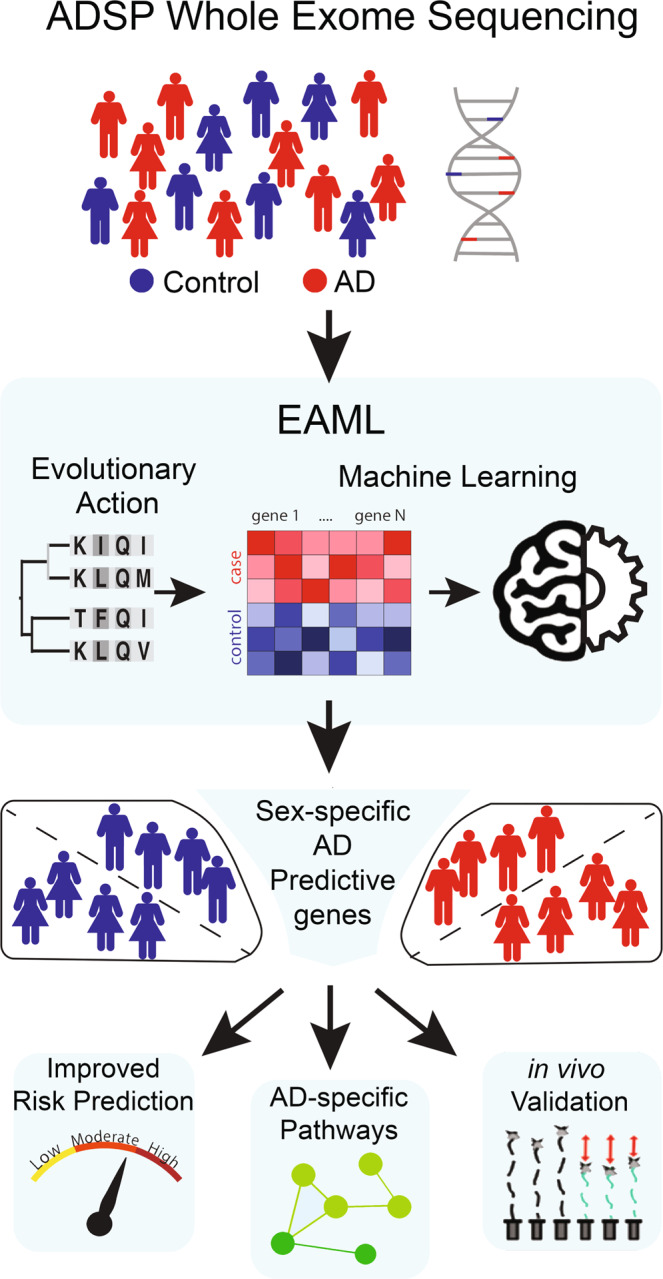
Schematic overview of study. This figure illustrates 4 steps of the study: ADSP WES data preparation, EAML run on the ADSP WES cohort, EAML runs of male and female separated cohort, and the criteria of success experiments to investigate predicted genes from EAML.

In order to identify novel sex-specific genetic drivers linked to AD, we developed a machine learning method that exploits whole exome sequencing (WES) data from the Alzheimer’s Disease Sequencing Project (ADSP)^18^ to identify genes that differentiate cases from controls. Figure 1 presents a graphical summary of this study. Unlike other approaches to this problem, our algorithm focuses on the functional impact of non-synonymous coding variants. These coding variants typically have unknown significance, but here we estimated their deleterious effect with the evolutionary action (EA) score^47^. For a given amino acid substitution at a given protein sequence location, this score is a product of the magnitude of the substitution times the functional sensitivity of the position. The former is estimated from amino acid substitution matrices. For example, an alanine to serine transition represents a small substitution magnitude while an alanine to tryptophan is a large one. The latter portion of EA is estimated from the evolutionary importance of each sequence position. For example, a position that varies often between phylogenetically close species is less sensitive than one that varies seldom and between phylogenetically distant species^48^. In this way, evolutionary action interprets the potential harm of human coding variants in light of past evolutionary divergences, providing consistently good performance with respect to other state-of-the-art methods and support in many practical applications. Notably, the evolutionary action score performed well in objective, blinded community challenges^48,49^ and was instrumental in suggesting candidate genes in autism spectrum disorder^50^, Alzheimer’s disease^51^, and cancer^52,53^.

Here for the first time, we combine evolutionary action with machine learning (EAML) by using it as a training feature to rank all genes in the genome for their ability to separate AD cases from controls. Strikingly, EAML maintains its accuracy in smaller samples and can be used separately on males and females to search for sex-specific AD genes. The genes we find are significantly involved in AD biology by multiple computational and experimental criteria and point to a cell-cycle/DNA repair module predictive of AD status specifically in females. These proof-of-concept findings support a general approach to identify genetic mechanisms linked to complex diseases by machine learning over case-control sequence data using phylogenetic evolutionary information. In AD, the results are new potential biomarkers for sex-sensitive diagnosis, drug development, and therapy.

## Results

### Learning AD-associated genes from the mutational impact of coding variants

To identify genes underlying LOAD we studied 2729 AD patients and 2441 control subjects from the ADSP cohort (dbGaP phs000572.v7. p4). Our ensemble computational approach, EAML, included nine separate machine learning methods, namely, PART^54^, JRip^55^, Multi-layer Perceptron^56^, Naive Bayes^57^, Logistic Regressions^58^, Nearest Neighbors^59^, Decision Trees Random Forest^60^, J48^61^, and Adaboost^62^. Each one used EA scores and the homo- or heterozygous status of coding variants of subjects (see Methods 4) to measure, with a Matthews Coefficient Correlation (MCC)^63^, how well each gene could separate patients from controls. After trying multiple aggregation metrics, including a voting system, cross-entropy-based ranking, and the MCC average, the average MCC over all nine algorithms was used to rank 17,400 human genes by their ability to predict AD and 98 genes met a significance cutoff (FDR < 0.01, Supplementary Data 1). The top gene was *APOE*, which suggested that EAML has the potential to identify AD risk genes.

### EAML genes are dysregulated in LOAD brains and enriched in pathways disrupted in AD

**Fig. 2:**
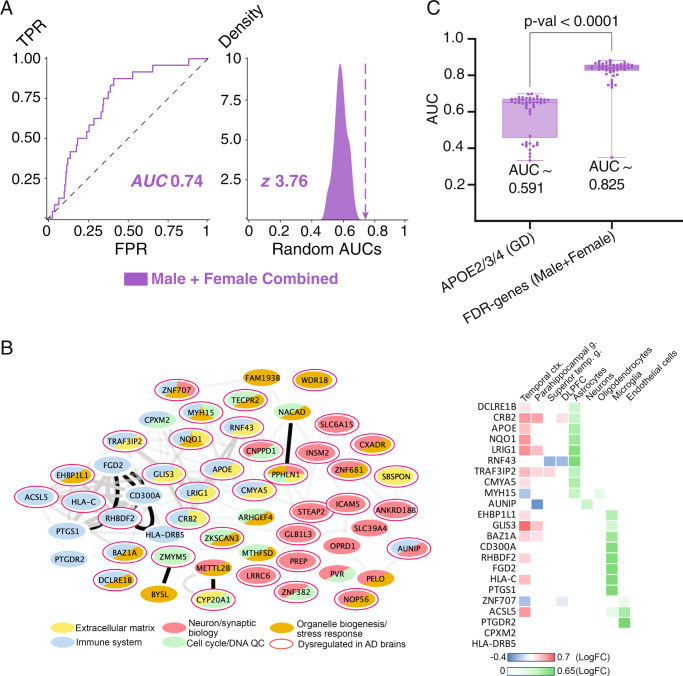
The top 98 EAML genes are connected to GWAS genes, dysregulated in AD patients, and capable of separating AD and healthy control samples. **A** Diffusions from the top 98 EAML genes from the full cohort to the 25 GWAS genes. AUC-ROC curve, *x*: False Positive Rate, *y*: True Positive Rate. This AUC-ROC curve represents the predictive power of 97 EAML genes to prioritize the 25 GWAS genes. Density distribution of randomly generated 100 AUCs based on randomly selected genes. The arrow indicates the AUC calculated from Fig. 2A. **B** (left) Integration of EAML candidates with expression networks dysregulated in AD. EAML candidates mapped into the AD consensus modules network (organic distribution) based on gene co-expression analysis^75,76,78^. The main function enriched in each module^75,76,78^ is indicated. Darker, thicker edges connect genes that are more highly correlated. Genes with a red ring are dysregulated in at least one brain region in AD vs control. (right) Heat map indicating which of the genes in the “immune system” module are dysregulated in AD brains. Also shown is the cell type in which their expression is enriched in the brain. **C** Risk prediction that based on the EAML genes for the full cohort. The box plot indicates minimum and maximum (lower and upper) whiskers, median (horizontal line), and first and third quartile (box). The mean AUCs of two groups (APOE vs. 98 FDR genes) and *p*-value of standard *t*-test (two sided) are shown.

In order to assess these 98 EAML candidate genes, we asked whether they were functionally connected with genes previously linked to AD. Label propagation in biological networks measures the functional proximity between two sets of genes^64–68^, and we computed propagation over the generic STRING v11 protein-protein interaction (PPI) network, first removing *APOE* from the 98 genes given its known connectivity to AD. To control for biases due to the high network connectivity of the EAML candidate genes, we also performed label propagation for 100 sets of 97 random genes with a similar distribution of degree connectivity. Compared to the random genes with equivalent connectivity, the 97 EAML candidates diffused significantly to twenty-five LOAD-associated genes from GWAS^17^ (z-score ~3.76 and AUC of 0.74, Fig. 2A and Supplementary Table 1). Additionally, previous studies show that genes and diseases whose keywords are co-mentioned in biomedical literature are likely to be biologically connected^69,70^ and the confidence of their associations is based on the number of papers with co-mentions. Thus, we repeated the diffusion analysis on a different network^71^ built from the keyword co-occurrences of genes, diseases, and drugs in biomedical papers from PubMed. The 97 EAML genes were significantly connected to AD genes (z-score ~6.2) and to dementia-related disorders compared to random disorders (z-score >3, Table 1). These data suggest that EAML genes are related to functional pathways enriched for AD genes and are consistent with roles in AD pathology and dementia.

**Table 1:** Diffusion (Z scores) from EAML genes to MeTeOR network

Entity Name	Sample Set		
	Combined	Male	Female
Alzheimer Disease	6.27	5.29	6.80
Dementia	1.84	1.58	1.88
Frontotemporal Dementia	1.49	0.71	0.60
Vascular Dementia	−0.14	0.48	0.44
Multi-Infarct Dementia	−0.15	−0.22	−0.07
AIDS Dementia Complex	−0.35	−0.35	−0.10

To further assess EAML candidates, we investigated whether they were connected to AD-related molecular changes. Leaving APOE aside again, we assessed the expression of the remaining 97 EAML candidate genes across AD brain data using the AMP-AD sequencing repository^72–77^. Forty-five EAML candidates were significantly dysregulated in AD patients versus controls in at least one brain region, suggesting that they either respond to or underlie AD-related insults and may therefore play a role in AD pathogenesis (Supplementary Fig. 1, hypergeometric test *p* = 0.04).

Next, we performed a functional enrichment analysis with respect to AD-related and brain-specific pathways using transcriptomic data from AMP-AD^72–78^ brain tissue. Various approaches have been developed to discover consensus transcriptional changes taking place in AD brains compared to controls. These efforts have led to the identification of several AD co-expression modules that are enriched in specific biological processes^75–78^. We mapped the EAML predicted genes onto these co-expression networks. We found that of the 53 EAML candidates that have been analyzed in the AD transcriptome, 22 belonged to modules related to immune response (Fig. 2B left, Fisher’s *p* < 0.005, consensus module B in^75,78^). Subnetworks within these immune modules revealed EAML candidates potentially involved in cytokine signaling (*HLA-C, ACSL5, PTGDR2, BAZ1A, DCLRE1B*), synapse pruning (*FGD2, RHBDF2*) and microglia pathogen phagocytosis (*EHP1L1, CD300A, FGD2, RHBDF2 and PTGS1*) (Supplementary Fig. 2). These functional enrichment results are consistent with the cell types in which these genes are expressed. The expression profile of these 22 genes in published snRNAseq^79,80^ datasets revealed that nine are enriched in astrocytes, 10 in microglia and three in endothelial cells, supporting their potential roles in neuroinflammation (Fig. 2B right). Among the three endothelial genes, the prostaglandin receptor *PTGDR2* stood out as we also identified the prostaglandin-endoperoxide synthase *PTGS1*, a key enzyme in prostaglandin production, which has been linked to AD pathology^81^. We also found enrichment in the immune system module when we integrated the 45 EAML candidate genes that were dysregulated in the AD brain transcriptome with the AD consensus modules (Fig. 2B left, consensus module A in^75,76,78^). These results support a role of many EAML candidates in neuroinflammation, microglial, and astrocytic biology. This is consistent with the observation that many AD-risk factors identified^17,18^ using different genetic approaches are involved in neuroinflammation and suggests that the EAML candidates may act through these same pathways.

### EAML candidates are modifiers of neurodegeneration

If EAML candidate genes belong to pathways that contribute to AD pathophysiology, we hypothesized that they would modify neurological phenotypes in vivo from two well-characterized *Drosophila* AD models expressing either secreted Aβ42 or wild-type human 2N4R tau^82,83^ specifically in neurons. Expression of either Aβ42 or tau in *Drosophila* leads to late-onset progressive neuronal dysfunction that can be accurately quantified using behavioral (i.e., motor performance) readouts. We used an automated system that video records animals as they climb a vial and uses their trajectories to calculate movement metrics such as speed^84^. This task provides a quantitative measure of motor performance that can be monitored longitudinally, as the animals age, and serves as an assay for neuronal dysfunction. We obtained loss of function, and shRNA strains available from public repositories targeting the *Drosophila* homologs of the 98 EAML candidate genes and tested each one in tau and Aβ42 *Drosophila* models (Supplementary Fig. 3A, B and Supplementary Data 2). In total we were able to test the homologs of 73 genes. We found that 36 of these genes modulated tau-induced degeneration when their function was decreased (12 worsened tau-induced degeneration while 24 ameliorated it). In the case of the secreted Aβ42 model, 17 genes were loss of function enhancers while 12 ameliorated Aβ42-induced neurodegeneration. These results represented a significant enrichment in genetic modifiers (Fisher’s test *p* = 0.0001 for tau modifiers and *p* = 0.0115 for the Aβ42 ones) when compared with the usual hit rate (between 15–20%) of our frequent unbiased genetic screens^82,85,86^. These results strongly connect the EAML candidates with the ability to modulate neurodegeneration in vivo. Importantly, we identified 27 genes whose knockdown attenuates neuronal deficits in vivo, highlighting their therapeutic potential.

### AD risk prediction

Since our EAML candidates arose from their individual ability to separate AD patients from controls, we reasoned that the combined genes set should perform well in predicting patient risk stratification. For this model, three efficient classifiers were retained: (1) Adaboost^62^ is an ensemble method that combines weak learners (e.g., decision stumps) into a stronger one with significant performance for binary classification; (2) Logistic regression^58^ is a less computationally intensive classifier that is particularly useful when relevant learning features are employed; (3) Random Forest^60^ is a decision tree-based model that effectively handles high dimensional genomic data. The three classifiers were then combined using a stacking approach^87^ supported by a decision tree algorithm in order to strengthen the performance of each individual method. Our predictive AD model was trained by 10-fold cross-validation, measuring predictive performance using the area under the curve (AUC) of the receiver operator characteristic. As a control, a similar model was built based on *APOE* genotype status plus age of onset information. This predictive model built with EAML candidates significantly outperformed the one built with *APOE* genotype plus age of onset (*t*-test *p* < 0.0001) (Fig. 2C). These data show that machine learning predictors trained with gene features selected by EAML perform better than *APOE* status and may have prognostic value for the risk of developing AD.

### EAML retains robust predictive power at small cohort sizes

**Fig. 3:**
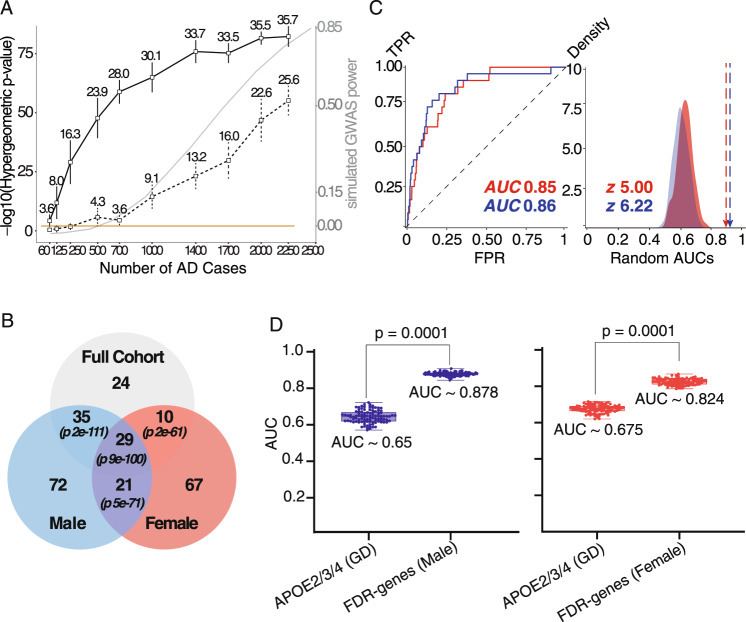
Down-sampling analyses and sex-separated analysis led to better risk prediction. **A** Down-sampling analyses, *x*: The number of randomly selected samples of AD cases; an equal number of control samples were also randomly selected. *y*: Hypergeometric *P*-values (one-tailed Fisher’s Exact test) comparing the top 50 genes from each iterated experiment to the top 50 genes from full cohort, solid-line: EAML, dot-line: SKAT-O; numbers indicates mean number of overlapped genes between each set and full cohort ADSP, the error bar indicates standard error for the mean number of overlapped genes; gray-line indicates simulated GWAS power using GWAS power calculator. **B** Venn-diagrams intersection between top EAML predicted genes from full cohort, male, and female. The number indicates the number of overlap genes between each EAML analysis. The hypergeometric *p*-value (one-tailed Fisher’s Exact test) was calculated using SuperExactTest R package^156^. **C** Diffusions from the top 157 genes from male (blue) and the top 127 genes from female (red) to the 25 GWAS genes. **D** Risk predictions that based on the EAML genes for male (blue), female (red). EAML significantly (*p* ~ 0.0001) improved prediction AUC comparing to APOE in male and female combined. When separating sexes, male’s genes significantly better predict than the combined (AUC 0.878 vs. 0.825) although female genes’ prediction AUC stay same (AUC 0.824 vs. 0.825). (*GD: APOE alleles + Age). The box plot indicates minimum and maximum (lower and upper) whiskers, median (horizontal line), and first and third quartile (box). The mean AUCs of two groups (APOE vs. 98 FDR genes) and *p*-value of standard *t*-test (two sided) are shown.

Given the strength of these associations between EAML genes and AD, we next sought to test the robustness of these findings through down-sampling. We compared gene candidates found by EAML when applied to sequentially smaller sub cohorts of randomly selected case and control subjects, starting from the full original 2729 cases versus 2441 controls down to 60 versus 60. For each different sample size, we performed independent EAML analyses on 10 different sets of randomly picked cases and controls (*n* = 10), resulting in 100 total experiments. For comparison, we ran in parallel the commonly used gene-based association analysis using SKAT-O^88^ (see Methods 3) on the same randomly selected cases and controls (10 iterations), and the same non-synonymous variants used in the corresponding EAML experiments. To assess performance in these down-sampled cohorts, we compared the top 50 candidates of 10 iterated experiments at each sample size from the EAML or SKAT-O results to the top 50 genes obtained from applying EAML or SKAT-O to the full ADSP cohort, calculating the Kendall-Tau ranking coefficient^89^ and hypergeometric overlap *p*-values to measure consistency. Strikingly, EAML produced consistent outputs and robust prediction capabilities at progressively smaller sample sizes. For example, at *n* = 700 AD cases (Fig. 3A), EAML identifies greater than 50% (hypergeometric *p* = *10*^*−58*^) of the candidates identified in the full cohort compared to 8% (hypergeometric *p* = *10*^*−4*^) identified by SKAT-O. Top EAML genes also ranked consistently across the decreasing sample sizes compared to the performance of the SKAT-O predictions at similar cohort sizes (Supplementary Fig. 4). Interestingly, the down-sampled hypergeometric *p*-value curve of SKAT-O was similar to the in-silico simulated GWAS power curve (gray line in Fig. 3A; see Methods 3). The simulated GWAS power decreased drastically as the sample size dropped (max: 0.85 at 2500, min: 0 at 250). Nine genes including *APOE* were recovered consistently by EAML (10 out of 10 iterations per sample size) across all conditions starting at 500 cases and 500 controls: *PRSS57*, *CPXM2*, *GJA3*, *PTGDR2*, *EHBP1L1*, *GZMA*, *DTL* and *GLB1L3* (Supplementary Data 3). Notably, most of these recurrent top genes have been linked to AD biology or are dysregulated in AD. *PRSS57* falls near the *ABCA7*^90,91^ locus. *CPXM2* regulates *CLU* levels and is linked to synaptic remodeling and several *CPXM2* SNPs are associated with LOAD^92–95^. *PTGDR2* is a receptor of prostaglandins, which play critical roles in inflammatory response and may contribute to AD^96–102^. *EHBP1L1* is an interactor of *BIN1*^17^ and its levels are increased in AD^103^. *GZMA* is a factor related to cytotoxicity following Herpesvirus infection and its levels are increased in effector memory T cells from AD patients^104^. *DTL* may mediate cell-cycle re-entry in AD neurons^105^ and *GLB1L3* is decreased in AD bulk brain tissue and single cells transcriptome (Supplementary Fig. 1). Taken together these data suggest that the power of EAML remains stable, consistent, and robust even at sample sizes too small for other methods, opening the possibility of interrogating smaller cohorts separating male and female to identify sex-specific modifiers.

### EAML identifies sex-specific LOAD-associated genes

In light of its robustness to small sample sizes, we searched for sex-specific AD related genes in the ADSP cohort by applying EAML separately to male (1215 AD cases + 1104 controls) and female (1514 AD cases + 1337 controls). EAML identified 157 and 127 top AD-associated genes in male and female, respectively (FDR < 0.01, Fig. 3B and Supplementary Data 1). In each of the sex-separated cohorts, we recovered *APOE* as the top hit. Next, we investigated whether the identified genes in the sex-separated EAML approach are connected to the 25 AD GWAS genes^17^ using the same network-based label propagation analyses performed above. Network diffusion of either the 157 male or the 127 female EAML candidates revealed robust and significant connectivity to the 25 GWAS genes over the STRING network (z-scores ~6.22 and ~5.0, Fig. 3C), and to Alzheimer’s Disease in the MeTeOR network (z-scores ~5.29 and ~6.79 for male and female, respectively, Table 1). Remarkably, connectivity for genes identified by the sex-separated EAML was significantly higher for both male and female (male z-score 6.22, female z-score 5.0, Fig. 3C) than in the sex combined approach (full cohort z-score 3.76, Fig. 2A). This increased connectivity of the sex-separated EAML candidates was also supported by the alternative prioritization approach, where sex-specific EAML candidates significantly prioritized the GWAS genes more than hits from the combined EAML (full cohort AUC of 0.74 in Fig. 2A, male AUC 0.86 and female AUC 0.85 in Fig. 3C). This increased network connectivity to the AD genes may not derive from the higher overlap of the sex-specific genes to the GWAS catalog loci since the network connectivity relies on STRING d/b while the overlap to GWAS catalogue loci depends on genomic locations. Taken together our results indicate that applying the EAML algorithm to sex-specific cohorts improves our ability to discover AD-related genes.

As with the full cohort, we next used the male and the female EAML candidates to perform sex-specific risk prediction. Even with half the cohort size, the ability to predict AD status was significantly improved in male (Fig. 3D left, AUC 0.878, *p* ≅ 0.0001) and was similar in female (Fig. 3D right, AUC 0.824, *p* ≅ 0.99) compared to full cohort (Fig. 2C, AUC 0.825). This further highlights the potential value of performing sex-specific analyses to improve the pre-symptomatic diagnosis of AD in male and female.

### EAML candidates from sex-separated cohorts identify pathways affected differentially in male and female

**Fig. 4:**
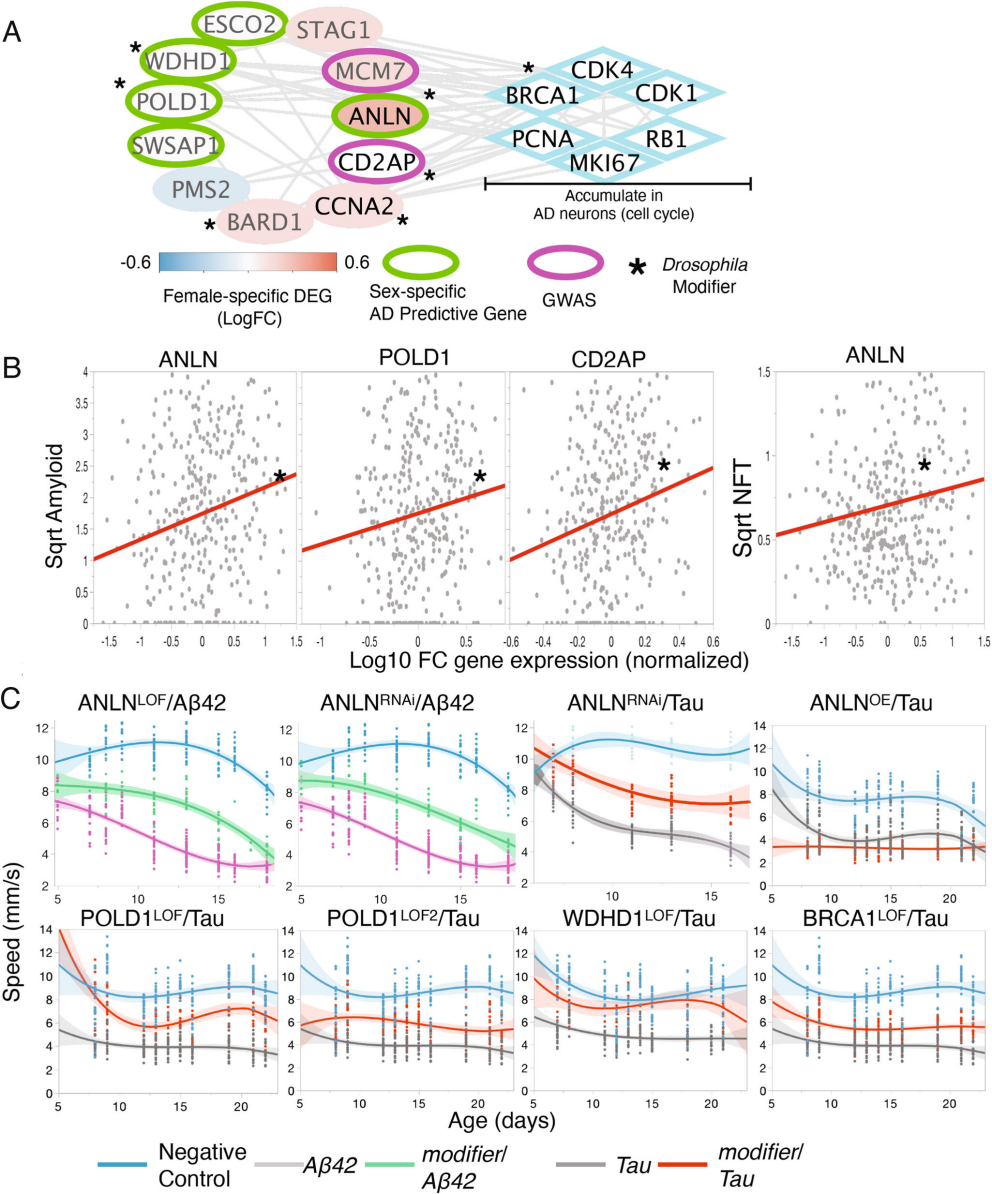
Characterization of a cell-cycle/DNA repair-associated module enriched in female-specific EAML candidates. **A** Integration of five female-specific EAML candidates involved in cell-cycle/DNA repair with genes predominantly dysregulated in female AD brains and cell cycle genes known to accumulate in AD neurons. **B** positive correlation of gene expression and neuropathologic features for some of the genes in the module shown in (**A**). **C** graphs representing longitudinal analyses of neuronal dysfunction assessed as speed of the animals as a function of age (days) for the indicated alleles. Blue corresponds to negative (healthy) controls expressing a non-targeting hp-RNA. Purple shows the performance of β42/ non-targeting and grey the performance of tau/ non-targeting hp-RNA diseased animals. Green or red show the performance of animals carrying the allele indicated on top and either expressing β42 (green) or tau (red) paneuronally. Knockdown of the *Drosophila* homologs of three of the EAML female-specific candidates (*ANLN*, *POLD1*, *WDHD1*) results in amelioration of the neurodegenerative phenotypes. Knockdown of the *Drosophila* homolog of *BRCA1* also ameliorates neuronal dysfunction (specific alleles used are indicated in Supplementary Data 2). Each graph shows the fit curve of the third-degree polynomial regression (dark line) and the confidence intervals for the same regression (shaded). All experiments shown are statistically significantly different *p* < 0.05 (exact *p* values are shown in Supplementary Data 2) when analyzed using non-linear random mixed effects model ANOVA. Four replicates of ten animals each per genotype were used for these experiments.

We sought to gain functional insights from the EAML candidates identified in the male and female cohorts. We integrated the EAML candidates with transcriptomic data from AD brains using the AMP-AD^72–78^ transcriptomic dataset to assess for enrichment in AD-related brain-specific pathways. As with the EAML candidates in the combined cohort, we observed a significant enrichment in genes belonging to the AD-consensus modules associated with immune response and extracellular matrix in both the male and female EAML candidates (Supplementary Fig. 5). We next explored the underlying biology of the EAML candidates that were identified only in the female cohort (female specific EAML genes), or only in the male cohort (male specific EAML genes). Integration of the male specific EAML candidates with the AD transcriptomic network revealed an enrichment in modules involved in stress response and organelle biology (*p* < 0.05, Supplementary Fig. 5A). On the other hand, integration of the female specific EAML candidates with the AD transcriptional signatures revealed an enrichment in modules associated with cell-cycle and DNA quality control (*p* < 0.05, Supplementary Fig. 5B). Prompted by this, we sought to identify specific pathways in which these male or female candidate genes may act. We used the STRING database to connect the sex-specific EAML candidates with sex-specific transcriptionally dysregulated genes from the AMP-AD database^72–78,106^. This effort did not highlight any additional modules in the male specific EAML candidates. However, this integration uncovered a module composed of several female specific EAML candidates that interact with genes dysregulated more frequently in female AD brains (Supplementary Fig. 6). Interestingly, this module included two genes previously linked to AD: *CD2AP*^15,107^ and *MCM7*^108^ (Fig. 4A). The main functional enrichment in this module was cell cycle control and DNA quality control. Among all the genes in the module, *ANLN* stood out as a female-specific EAML candidate and is dysregulated at the transcriptional level only in female AD cases. Furthermore, expression of *ANLN* and *POLD1* correlated with AD neuropathology in human brains (Fig. 4B). Accumulation of cell cycle markers in post-synaptic neurons is a hallmark of AD and other neurodegenerative diseases^109–119^. Interestingly, a large number of cell cycle/DNA quality control genes that accumulate in AD neurons are direct STRING interactors of genes identified in the female specific EAML module. Prompted by this, we investigated whether the EAML candidates in this module and their interactors could modulate neuronal dysfunction associated with AD. We tested the *Drosophila* homologs of the genes in this module and their interactors (Fig. 4B) for their ability to modify Aβ42 or tau-induced neurotoxicity using loss of function and overexpression alleles. Decreasing the expression of 3 female specific EAML candidates (*ANLN*, *POLD1* and *WDHD1*) resulted in amelioration of Aβ42 and/or tau-induced neuronal dysfunction in vivo (Fig. 4C). Knockdown of the *Drosophila* homologs for three other genes in this module (*BARD1*, *CCNA2* and *CD2AP*^120^) also modulated neuronal dysfunction in *Drosophila*. Among the cell cycle genes that accumulate in AD neurons, *BRCA1* stood out in this module as it interacts with *ANLN* and *BARD1*, two genes whose modulation ameliorates neurodegeneration. Therefore, we tested the effect of knocking down the *Drosophila* homologs of *BRCA1* o`n neurodegeneration. We found that reducing expression of BRCA1 *Drosophila* homologs significantly attenuated tau-induced neuronal deficits (Fig. 4C). Taken together, our results support a role for these cell-cycle and DNA quality control genes in AD pathogenesis, but more importantly they also suggest that these genes play a different role in female versus male, as they are more strongly associated to AD risk in female than male.

## Discussion

Sex-specific differences in brain physiology and function are beginning to emerge and may underlie the differential predisposition to CNS diseases such as autism and depression between male and female^46,121^. Although sex differences greatly impact AD risk, the specific effect of sex has been largely ignored in the context of AD genetics^28^. In this study, we developed a methodology that specifically targeted the genetic factors that influence AD risk separately in males and females. We were able to achieve this goal by combining machine learning with evolutionary data in the form of EA scores that predict the effect of coding mutations on protein function. This EAML framework proved robust with sufficient predictive power at small sample sizes to examine samples of male and female separately. EAML identified numerous AD-associated genes not found in the combined sex cohort (Fig. 3B). Remarkably, the sex-separated analyses had better connectivity to known GWAS genes (z-scores ≅ 6.22 and ≅ 5.0 for male and female, respectively, Fig. 3C) than candidates derived from the combined cohort. This suggests that separating the cohort by sex produced a more sensitive analysis. A total of 50 EAML candidates overlapped between male and female (Fig. 3A, p 5 × 10^−71^). Interestingly, 21 of these were not identified by applying EAML to the combined cohort (Fig. 3A). Five of these 21 genes (*PTPLA, ABI3, OR5AC2, MAPT, ECE2*) have previously been associated to AD risk by other groups^122–126^, reinforcing that EAML is effective in identifying AD-associated genes and highlighting the increase in sensitivity upon sex separation. Importantly, EAML also uncovered sex-specific candidates associated with AD risk in female but not male and vice versa (Fig. 3A), suggesting that certain biological pathways may play a greater role in AD for one sex than the other. In line with this, we found that while neuroinflammation was the most enriched process among the EAML candidates identified in the combined cohort, the picture changed once we focused on sex specific EAML genes. We found genes associated with stress response and organelle biology were enriched in male specific EAML hits but found cell-cycle and DNA quality control/replication genes in female specific hits. Since cell cycle markers are known to accumulate in AD patients, we followed up on this set of genes. Five female specific EAML candidates (*ESCO2, WDHD1, POLD1, SWSAP1* and *ANLN*) clustered together with genes whose expression is dysregulated predominantly in female AD brain. These genes correlated with neuropathological hallmarks of AD and modulated tau/Aβ42-induced neuronal dysfunction in vivo. Even though dysregulation of cell-cycle proteins and their abnormal aggregation in AD neurons has been previously reported^109–119^, our findings suggest that this pathway may contribute more to AD risk in females than males.

The above findings could have implications in how AD therapeutic strategies are developed and implemented. Of all the genes identified in this study, eleven have drugs that have been characterized as agonists or antagonists of their function (*PTGDR2, SLC6A15, OPRD1, PSMF1, NQO1, GJA3, DDR1, TPO, PTGS1, POLD1, RET*). Interestingly, of a total 97 compounds that target these genes, 40 have co-mentions with AD in PubMed. Of the 11 genes, *GJA3* and *DDR1* are EAML AD predictors in male only. Interestingly, the DDR1 inhibitor nilotinib is currently being investigated in a clinical trial for AD^127^ while a second DDR1 inhibitor (imatinib) has shown protective effects in mice^128^. On the other hand, four genes with known pharmacological agents (*TPO, PTGS1, POLD1* and *RET*) are EAML AD predictors in female but not male. RET stands out in this group, as three of its inhibitors - sunitinib^129^, imatinib^128^ and regorafenib^130^ - have shown beneficial effects in mouse AD models. The data presented here supports the stratification of clinical trials based on sex and indicates that some strategies may be more effective in female or male due to sex specific genetic risk factors.

EAML also improved risk prediction ability. Early diagnosis of AD risk based on genomic profiles would be a significant advance as patients will likely require early intervention to avert dementia^131,132^. Current risk prediction for AD using Polygenic Risk Scores (PRS) ranges from 60^133^ to 80%^134,135^ accuracy. This needs to be improved before the results can be used clinically as many of the loci belong to non-coding regions. Recently, ML approaches have gotten attention for risk prediction in complex diseases^136^ due to their ability to evaluate non-linear genotype-phenotype associations and their interactive effects^137^. The combination of EAML candidates with their EA profile as features for ML resulted in great accuracy at predicting AD risk (*AUC* 0.825, Fig. 2C). Remarkably, the sex separated EAML candidates did not exhibit a decreased prediction accuracy even though we used half of the cohort (Fig. 3D). Furthermore, that accuracy was significantly improved in the case of male (*AUC* 0.878, Fig. 3D left), highlighting the importance of sex-separated genomic analysis in AD risk prediction approaches.

Of note, we did not perform hyperparameter tuning and optimization with each individual ML algorithm and instead we used the preset parameters. Tuning can improve ML prediction accuracy. However, if done inadequately, tuning can lead to overtraining with poor performance on test data different from the training sets. This general contest between optimization and overtraining currently pervades ML approaches. In order to add robustness to the ML predictions we took several steps. First, rather than optimizing each individual algorithm, we ranked the hit genes based on their reproducibility across classifiers, thus attenuating the risk of artefactual overfitting in one specific algorithm. Second, additional confidence in the hit genes came from performing ten-fold cross validation with each classifier. Importantly, we optimized the quality of the information we input to the off-the-shelf classifiers by incorporating functional mutational information. We reasoned these measures will decrease the likelihood of selective overtraining and instead increase our method’s likely range of validity to data other than those trained upon. Our confidence in the resulting hit genes is supported by multiple independent criteria for success that all show that our candidate genes are reliably linked to AD, experimental evidence showing candidate genes modulate neurodegeneration in a live animal model and the ability of the hit genes to stratify patients. These independent lines of evidence suggest that parameter tuning was not a significant issue in this study, and it is likely not weakening the findings. We speculate that the theoretical advantage of optimizing parameters may not warrant the risk of overfitting that this would entail. We will explore this possibility in future studies. Additionally, we did not take into account synonymous variants or those affecting non-coding DNA regions, epigenetic phenomena such as methylation and histone modification, and we also limited to European ancestry. These additional sources of data would be complementary to our findings since studies have shown that different genetic ancestries play important role in AD^138,139^ as well as non-coding DNA variations^140^ and epigenetic efects^141^. As more and larger cohorts become accessible, we will expand the EAML approach to additional ancestries, other interesting subgroup comparisons such as stratifying by comorbidities or presence of certain neuropathological features as well as hyperparameter tuning of each ML algorithm to improve performance without overfitting.

In summary this work indicates that application of machine learning approaches to WES and WGS increased our resolution for AD risk genes compared with current standard statistical methods (e.g., SKATO). These data offer a proof-of-concept for the combination of evolutionary information and phylogenetic speciation with case-control sequencing data to identify genetic mechanisms linked to complex diseases. Importantly, focusing the study on sex-specific sub-cohorts increased our resolution to identify disease-related genes. This emphasizes the need to systematically apply sex separation to disease-gene association analyses, beyond analyzing the combined cohorts. Our pipeline has identified a significant number of AD-associated genes with sex-sensitive pre-symptomatic diagnostic power and potential therapeutic value that we will follow up on in pre-clinical studies. As larger sequencing datasets become available, application of EAML will help generate a more complete picture of the different sex-specific mechanisms involved in AD and other polygenic disorders.

## Methods

This study protocol (H-37394) was approved by the Institutional Review Board for Human Subject Research for Baylor College of Medicine and Affiliated Hospitals (BCM IRB). The information about informed consent from the study participants can be found in the study homepage (https://www.ncbi.nlm.nih.gov/projects/gap/cgi-bin/study.cgi?study_id=phs000572.v7.p4#restricted-access-section).

### Whole exome sequencing data

We obtained whole exome sequencing (WES) data from the Alzheimer’s Disease Sequencing Projects (ADSP) Discovery cohort from dbGaP (phs000572.v7.p4). In total, we analyzed 2729 Alzheimer’s Disease cases and 2441 healthy control white samples.

### Quality controls (QC)

We carried out QC processes to identify potentially false-positive variants and outlier samples. We calculated the Ti/Tv and the number of variants in different variant classifications such as non-synonymous, synonymous as well as the total number of variants and singletons. HWE (Hardy Weinberg Equilibrium) exact test^142^ was performed on the control samples of each cohort. We counted the ratio of heterozygotes and homozygotes in the sex chromosome to compare to the self-reported sex. Then we filtered out non-European descendants. We used Annovar^143^ to annotate the consequences of variants. We focused on the non-synonymous single nucleotide variants (SNVs) and small indels, which lead to the loss of functions of genes, excluding CNVs (copy number variants). We used PCA (Principal Component Analysis) to cluster the genetic background and identify outliers within the cohort samples. We inferred the genetic relationships between each sample by estimating the kinship coefficients and IBD (Identical by Descent). We used BCFTOOLS^144^ and PLINK^145^ for variant filtering and statistics, KING^146^ for inferring relationships, and SMARTPCA from Eigenstrat package for PCA^147^.

### GWA (genome wide association) analysis and power simulation

We performed variant-wide GWA for common variants (MAF ≥ 0.05) and gene-based association test for rare variants (MAF < 0.05). The *APOEε4* variant (rs429358) yielded genome-wide significant *p*-values 10^−20^ without covariate *APOEε4* status. With covariates sex, *APOEε4* status, PC1 and 2, only *TREM2* gene yielded a significance (*p* < 5 × 10^−6^) in ADSP. In the variant levels of *TREM2* gene, we found 50 alleles in AD cases versus 9 in controls (Fisher’s Exact *p* = 2.1 × 10^−7^) for the previously known missense variant p.R47H^148,149^.

We performed association analyses for non-synonymous variants, which were used in the EAML analyses. For a common variant (≥ minor allele frequency 0.05), we carried out variant-wide association test including the covariates - sex, age, *APOEε4* allele counts, and the first and second principal components from PCA to correct the effects of sex, age, ethnic differences and *APOEε4* status using the logistic-regression function in PLINK software. We used the SKAT-O test^88^ in EAPCTS analysis package (https://genome.sph.umich.edu/wiki/EPACTS) to test gene-wide associations for burdens of rare variants (minor allele frequencies <0.05). The GWAS power calculated on GAS power calculator (http://csg.sph.umich.edu/abecasis/cats/gas_power_calculator/) assuming Significance Level: 5E-6, Prevalence: 0.01, Disease Allele Frequency: 0.05, Genotype Relative Risk: 1.6.

### EAML framework

The first step measures the impact of the amino acid on overall fitness. Rather than attempt to compute this effect stepwise on successive but poorly characterized features, such as protein folding, dynamics, expression, translation, and myriad interactions among diverse components of multiple pathways, we modeled all these perturbations in aggregate through a formal evolutionary fitness (potential) function *f* that maps genotypes *γ* to points in the fitness landscape *φ*. Assuming, as a hypothesis, that *f* exists and is differentiable, a single coding perturbation *dγ* then has an impact given by:

1 $$\nabla f\cdot d\gamma=d\varphi$$

where, as shown before^47^, ∇*f* is the gradient of *f* approximated with the Evolutionary Trace algorithm^150^, and *dγ* is the magnitude of a single missense substitution approximated with amino acid substitution log-odds.

#### Gene-level metric

The underlying basis of our approach rests on the ability to amalgamate all variant level effects into one metric at the gene level. To do this, we developed a novel scoring system which uses EA at its core, dubbed EA probability or (pEA). This is defined as:

2 $$\left\{\begin{array}{c}0\,{iff}\,{silent}\,{mutation}\\ {x}_{{EA}\, > \,C}^{i}=1-{\prod }_{j=1}^{k}{\left(1-{{EA}}_{j}/100\right)}^{{zygo}}\,\forall {EA} \, > \,C\\ 1,\,{iff}\,{stop}\,{or}\,{indels}\,{mutations}\end{array}\right.$$

where k is the total number of variants in an individual, and EA_j_ is the Evolutionary Action scores from (1) of a given variant. This allows us to estimate the complete mutational effect of all variants within a given gene for a given individual.

#### Feature development and design matrix architecture

By presuming no information about a gene beforehand we allow the possibility that any given gene can affect the phenotype in either an autosomal dominant or recessive manner. We also presume that different levels of EA correspond to different magnitudes of fitness effects. These levels are as follows: EA > 1, EA > 30, EA > 70. In doing this, we can separately address the impact of variants above a given EA threshold. To develop features on which our framework will learn, we combine these different levels and manners of inheritance into six features of: Autosomal Dominant & EA > 1, Autosomal Dominant & EA > 30, Autosomal Dominant & EA > 70, Autosomal Recessive & EA > 1, Autosomal Recessive & EA > 30, Autosomal Recessive & EA > 70. We define autosomal dominant features to include all variants of a gene which are either heterozygous or homozygous, while recessive features only consider variants that are homozygous. This allows us to strictly address recessive features with only homozygous variants, while still considering all variant effects in the dominant features. Finally, these features are aggregated into a design matrix of the architecture *n x p* where n is number of samples and *p* is (6 features × number of genes). The genes tested come from the canonical RefSeq gene set.

#### Sub-setting of design matrix for 10-fold cross validation

To avoid overfitting and determine key driver genes with the highest prediction accuracy, we used a 10 folds cross validation (10-CV) method. The cohort was separated into 90% training data to fit the classification models and 10% testing data to validate the predictions. This process was repeated 10 times, shuffling the training and testing data sets.

#### Machine learning architecture

Our learning architecture consists of 9 different classifiers. Exploiting the uncertainty in linear and nonlinear genotype-to-phenotype relationships, these classifiers include Association Rules (PART^54^, JRip^55^), Function Optimizations (Multi-layer Perceptron^56^, Naive Bayes^57^, Logistic Regressions^58^, and Nearest Neighbors^59^), Decision Trees (Random Forest^60^ and J48^61^), and Meta Classifiers (Adaboost)^62^. This architecture was implemented in Weka (https://www.cs.waikato.ac.nz/ml/weka/). In MultiLayerPerceptron classifier, we used backpropagation with learning rate of 0.3, momentum of 0.2 and 4 hidden layers. In PART classifier, the confidence threshold for pruning is set to 0.25 and the minimum 5 objects per leaf is used. In Random Forest classifier we used 10 trees. In JRip classifier we used 3 folds, where one fold is used as pruning set, minimal weights of instances within a split is set to 2, with 2 runs of optimization. In J48 classifier, confidence threshold for pruning is set to 0.25 with the minimum of 2 instances per leaf. In Naïve Bayes classifier we used kernel density estimator. In Logistic Regression classifier we used Ridge for the regularization. In KNN classifier we set k = 3, using Euclidean distance with linear nearest neighbor algorithm. In Adaboost classifier, we used decision stump as the base learner.

#### Evaluating independent gene-level associations

Notably, our framework allows us to evaluate each gene in the context of the phenotype independently from effects of other genes. To evaluate the predictive power of an individual gene, we use a Matthew’s Correlation Coefficient (MCC) defined as:

3 $${MCC}=\frac{{TP}\,x\,{TN}-{FP}\,x\,{FN}}{\sqrt{({TP}+{FP})({TP}+{FN})({TN}+{FP})({TN}+{FN})}}$$

This measurement is utilized due to its robustness when considering imbalanced class sizes.

### Prioritization of top EAML predictions for further analyses

To prioritize EAML prediction genes for further analyses, we selected genes with an FDR corrected p-value less than 0.01, which is derived from the MCC distribution. These thresholds resulted in 98, 157, 127 genes from male/female full cohort, male, and female, respectively (Supplementary Data 1).

### Risk prediction (classifier)

EAML successfully identifies several genes which have been demonstrated to be quantitatively and biologically related to AD pathophysiology. However, EAML identifies these genes in the context of their individual and independent effects on disease. In order to understand the combined effect of the EAML predicted genes on disease status, we designed an experiment to test predictive power of these genes. In other words, can we combine the genes, identified for their individual influence in distinguishing affected versus healthy, to create a powerful predictor of disease status? We further extend this question by asking if we can create predictive models that are sex specific. In all three cases (cohort wide, male specific, female specific) the predictor would stratify an individual into a phenotypic class (healthy or affected) by learning some arbitrary function over the combined mutational profiles of the individual’s genes, specifically the EAML predicted genes. In order to determine whether the predictive power of the EAML genes is superior to traditional predictive factors, a second predictive model was developed based on the APOE genotype status, which is independently genotyped, and normalized age of onset of an AD individual.

In order to build these predictive models, input data must first be reformatted. For predictive models based on EAML genes, this reformatting was done as follows. Consider any one sample *n* out of *N* samples and *h* EAML predicted genes, for the purpose of a predictive model this sample will be represented as a vector of *h* elements, where each element is given by the pEA of particular EAML gene: *n* = [*pEA*_1_, *pEA*_2_,...,*pEA*_*h*_] where *pEA*_*i*_ is the *pEA* value of ${gen}{e}_{i}$. Each sample *n* is therefore represented as a vector of dimensions 1x*h*. For *N* samples in a cohort, we then have an input matrix of size *N*x*h*. Similar processing is done for all male specific, female specific, and cohort level predictive models.

Similar reformatting was done for predictive models based on *APOE* genotype status and the age of onset, which were also used in the complementary GWAS (see Methods 3). Each individual in the cohort was represented as a 1 × 2 vector where the first element corresponds to *APOE* variant status, and the second element of the vector is the age of onset. *APOE* variant status of an individual was represented as the pEA value for the individuals *APOE* variants. For example, if an individual had *APOE3*/*APOE3* (pEA = 0) variant and age of onset was 64, they were represented as *n* = [0,64]. For *N* samples in a cohort, we then have an input matrix of size *N*x2. After the cohort level matrix is calculated, normalization is done for age of onset features by subtracting the mean and dividing by standard deviation so that age of onset feature is on a similar scale as pEA features, this is a commonly used practice in machine learning to standardize and scale input data. Similar preprocessing was performed for all male specific, female specific, and cohort level predictive models.

In order to fairly compare and contrast the predictive models, the same machine learning architecture was used for all six predictors. This architecture was an ensemble learner composed of three different classifiers: random forest, logistic regression, and adaboost. Ensemble learning, in particular stacking, was used because stacked ensemble learning models have been shown to be a simple yet effective method for improving predictive accuracy compared to individual classifiers used separately^151–153^. Random forest, logistic regression, and adaboost were chosen as the ensemble classifiers due to two main characteristics. First, out of the nine classifiers used in the EAML architecture, these three demonstrated consistent and high MCC predictions. Second, these three classifiers were empirically identified to train and converge the fastest out of the nine EAML classifiers. Finally, in order to learn how to combine predictions from the three classifiers into one final prediction for a sample, we use a decision tree-based algorithm (Hoeffding tree) to train the individual classifiers together. Here, the Hoeffding tree serves the purpose of computing how much weight to give a specific classifier when making the final prediction, e.g., 30% of the final decision may be based on logistic regression prediction, 30% from adaboost prediction, and 40% from random forest prediction. All classifiers were trained using default hyperparameters and regularizers. The split decision was set to 1e-6, the minimum fraction of weight for info gain splitting was fixed to 0.01 and finally two grace periods of resp. 200 and 300 were considered. Implementation of the complete architecture and learning schemes, like EAML, were done using WEKA package for machine learning.

Complete training of the architecture is done through a k-fold cross validation approach. In this approach, the cohort is split into *k* equal sub-cohorts, and one of the *k* sub-cohorts is set aside for testing while the other *k*−1 sub-cohorts are used for training. This procedure is repeated *k* times such that each sample in the cohort is both trained on and tested on independently. This is a commonly used approach in machine learning as it is a particularly good preventative measure against model overfitting. For our implementation, we use **k** = 10 folds. Predictive power of the models is measured as area under curve (AUC) for receiver operator characteristic. AUCs are reported per test set of each of the k-folds. Finally, to compare between model performance using EAML predicted genes and *APOE* variant + age, *t*-tests were performed between the distribution of AUCs seen in both types of models and the corresponding *p*-values are reported.

### Network analyses

We performed graph-based diffusion (GID) method^64–66^ in order to evaluate how well the 98 EA-ML predictions are connected to manually curated AD gold standard genes 25 GWAS genes and to dementia-related disorders in biological networks.

STRING network version 11.0 was downloaded from http://version11.string-db.org. We used the combined score that cover evidence from all sources. The network contains 19,247 genes and gene products, in which 98 EAML genes are present. Graph-based information diffusion (GID) method^64–66^ was applied to measure how well two groups of genes are connected to each other. Through GID, functional information was propagated from genes of interest to all genes in the network through their connections. Genes receiving significantly more diffusion signals than random genes are more connected and, thus functionally related to the original genes. Signals were diffused from one group or their comparative random sets to another group. Random genes were also selected from other genes in the network and had similar degrees of connectivity with predicted genes that initiate signals. We validated whether the AD known genes receive significantly more diffusion signals from the predicted AD genes than other genes in the network, and vice versa through area under the curve (AUC) for receiver operating characteristic (ROC). For a diffusion experiment of each pair of predicted groups, random was performed 100 times to obtain a distribution of random AUCs, which was tested for normality. Z-score, which is the number of standard deviations from the random mean, was computed for the experimental AUC based on the distribution of the random AUCs.

A literature network, called MeTeOR^71^, was used to explore potential literature relatedness of the predicted genes with dementia-related disorders. MeTeOR aggregates publication co-occurrences of Medical Subject Headings (MeSH) terms, which are manually curated by PubMed to annotate key topics, genes, diseases, and chemicals of given articles. Biological entities that are co-mentioned together in publications are more likely to be functionally related. MeTeOR has previously been utilized to explore known and novel meaningful biological associations^70,71^. We performed the GID method to evaluate whether the predicted 98 genes are well connected to dementia-related disorders in the MeTeOR literature network and thus, are likely to be involved in dementia pathology. Dementia-related disorders were selected from disease terms annotated in MeTeOR that consist of “dementia” and “Alzheimer”. This approach yielded 6 terms for dementia-related disorders Table 1). We compared the diffusion signals that each of the 98 genes received from the 6 dementia-related disorders against random genes that matched the connectivity degrees with the genes in the MeTeOR network. We also evaluated diffusion signals for each dementia disorders from the 98 genes against random. We computed z-scores to compare diffusion signals of the predicted genes and dementia disorders against random. Z-scores above 2.5 were considered significant.

For the coexpression analysis of single cell RNAseq, cell type specific WGCNA networks were obtained from^154^. Using Cytoscape, we identified the primary degree coexpressed nodes for the EAML genes and built coexpression communities using the *HiDef-Louvain* algorithm tool in the *Community Detection* extension. We obtained ~100 communities for each cell type, and then run the functional enrichment tool in the *Community Detection* extension to explore functional overlap in gProfiler, enrichR and iQuery databases applying an FDR *q* < 0.05.

For the coexpression analysis of the AD-specific coexpression networks, we used the AD- coexpression networks built in^76^ and used the first-degree nodes between genes as edges to map the EAML genes into the different functional modules. Enrichment was calculated using Fisher’s test and considered significant if *p* < 0.05.

### Experimental integration

#### *Drosophila* strains and motor performance assay

Genetics and strains: the *Drosophila* lines used to drive expression of either wild-type human 2N4R tau (*UAS-Tau*) or secreted β_42 (_*UAS−Aos*:β42) were previously reported^82,83^ and are available from the Bloomington *Drosophila* Stock Center (BDSC), University of Indiana. For pan-neuronal expression we used the *elav-GAL4C155* driver obtained also from BDSC. The alleles and shRNAs tested as candidate modifiers were obtained from the BDSC or from the Vienna *Drosophila* Resource Center (VDRC).

#### Motor performance assays

To assess motor performance of fruit flies as a function of age, we used ten age-matched females per replica per genotype as previously described^84^. Flies are collected in a 24 h period and transferred into a new vial containing 300 μl of media every day. Tau animals were kept at 23 C while β_42_ experiments were maintained at 28 C. Four replicates were used per genotype. Using an automated platform, the animals are taped to the bottom of a plastic vial and recorded for 7.5 s as they climb back up on the walls of the vials. Videos are analyzed using custom software to assess the speed of each individual animal. Four trials per replicate are performed each day shown, and four replicates per genotype are used. Using the average performance of all 10 animals in each replicate and 4 replicates per genotype, a nonlinear random mixed effect model ANOVA^155^ was applied to the average using each four replicates to establish statistical significance across genotypes. Specifically, we looked at differences in regression between genotypes (genotype *p* value) and also between genotypes with time (additive effect, represented by a shift in the curve, genotype+time *p* value). *P*-values were adjusted for multiplicity using Holm’s procedure. Code for this analysis is available upon request from the Botas Laboratory. All graphing and statistical analyses were performed in R. The non-targeting shRNA line V2691 from the VDRC was used to generate negative controls (*Elav-GAL4/UAS-V2691*) to establish the healthy baseline motor performance and disease controls (either *Elav-GAL4/UAS-Tau/UAS-V2691* or *Elav-GAL4/UAS-Aos:β42/UAS-V2691*) for the disease baseline.

### Reporting summary

Further information on research design is available in the Nature Portfolio Reporting Summary linked to this article.

## Supplementary information

Supplementary Information

Description of Additional Supplementary Files

Supplementary Data 1-3

Reporting Summary

## Data Availability

The ADSP Whole Exome Sequencing data used in this study are available in the dbGaP database under accession code phs000572.v7.p4. The raw WES data are protected and are not available due to data privacy laws. Evolutionary Action scores of missense variants are publicly available via web server (http://eaction.lichtargelab.org/). The AMP-AD transcriptomic data used in this study are available in the AD knowledge portal (https://adknowledgeportal.synapse.org/) database under accession code syn8484987, syn8466812, syn8456629. The EAML candidates’ genes and its fly test results generated in this study are provided in the Supplementary Information/Data file. All data supporting the findings described in this manuscript are available in the main article file, the supplementary Information, the supplementary file, or by corresponding author upon request.

## Code availability

The EAML code can be downloaded from GitHub (https://github.com/LichtargeLab/EAML) including an easy installation guidance and a toy dataset. The code for the motor performance analysis is available upon request from the Botas Laboratory (Ismael.alramahi@bcm.edu or jbotas@bcm.edu).

## References

[1] Van Cauwenberghe C, Van Broeckhoven C, Sleegers K (2016). The genetic landscape of Alzheimer disease: clinical implications and perspectives. Genet. Med..

[2] Chartier-Harlin MC (1991). Early-onset Alzheimer’s disease caused by mutations at codon 717 of the β-amyloid precursor protein gene. Nature.

[3] Goate A (1991). Segregation of a missense mutation in the amyloid precursor protein gene with familial Alzheimer’s disease. Nature.

[4] Janssen JC (2003). Early onset familial Alzheimer’s disease: mutation frequency in 31 families. Neurology.

[5] Campion D (1995). Mutations of the presenilin I gene in families with early-onset alzheimer’s disease. Hum. Mol. Genet..

[6] Rogaev EI (1995). Familial Alzheimer’s disease in kindreds with missense mutations in a gene on chromosome 1 related to the Alzheimer’s disease type 3 gene. Nature.

[7] Sherrington R (1995). Cloning of a gene bearing missense mutations in early-onset familial Alzheimer’s disease. Nature.

[8] Gatz M (2006). Role of genes and environments for explaining Alzheimer disease. Arch. Gen. Psychiatry.

[9] Karp A (2006). Mental, physical and social components in leisure activities equally contribute to decrease dementia risk. Dement. Geriatr. Cogn. Disord..

[10] Roe CM, Xiong C, Miller JP, Morris JC (2007). Education and Alzheimer disease without dementia: Support for the cognitive reserve hypothesis. Neurology.

[11] Sando SB (2008). Risk-reducing effect of education in Alzheimer’s disease. Int. J. Geriatr. Psychiatry.

[12] Wang HX, Karp A, Winblad B, Fratiglioni L (2002). Late-life engagement in social and leisure activities is associated with a decreased risk of dementia: a longitudinal study from the Kungsholmen Project. Am. J. Epidemiol..

[13] Guerreiro R (2016). Genome-wide analysis of genetic correlation in dementia with Lewy bodies, Parkinson’s and Alzheimer’s diseases. Neurobiol Aging.

[14] Gatz M (1997). Heritability for Alzheimer’s disease: the study of dementia in Swedish twins. J. Gerontol. - Series A Biol. Sci. Med. Sci..

[15] Lambert JC (2013). Meta-analysis of 74,046 individuals identifies 11 new susceptibility loci for Alzheimer’s disease. Nat. Genet..

[16] Jansen IE (2019). Genome-wide meta-analysis identifies new loci and functional pathways influencing Alzheimer’s disease risk. Nat. Genet..

[17] Kunkle BW (2019). Genetic meta-analysis of diagnosed Alzheimer’s disease identifies new risk loci and implicates Aβ, tau, immunity and lipid processing. Nat. Genet..

[18] Bis JC (2018). Whole exome sequencing study identifies novel rare and common Alzheimer’s-Associated variants involved in immune response and transcriptional regulation. Mol. Psychiatry.

[19] Ridge PG, Mukherjee S, Crane PK, Kauwe JSK (2013). Alzheimer’s disease: analyzing the missing heritability. PLoS One.

[20] Corder EH (1993). Gene dose of apolipoprotein E type 4 allele and the risk of Alzheimer’s disease in late onset families. Science (1979).

[21] Corder EH (1994). Protective effect of apolipoprotein E type 2 allele for late onset Alzheimer disease. Nat. Genet..

[22] Neu SC (2017). Apolipoprotein E genotype and sex risk factors for Alzheimer disease: a meta-analysis. JAMA Neurol..

[23] Beydoun MA (2012). Sex differences in the association of the apolipoprotein E epsilon 4 allele with incidence of dementia, cognitive impairment, and decline. Neurobiol. Aging.

[24] Mortensen EL, Høgh P (2001). A gender difference in the association between APOE genotype and age-related cognitive decline. Neurology.

[25] Sundermann EE (2016). Female advantage in verbal memory: evidence of sex-specific cognitive reserve. Neurology.

[26] Sundermann EE (2016). Better verbal memory in women than men in MCI despite similar levels of hippocampal atrophy. Neurology.

[27] Sundermann EE (2017). Does the female advantage in verbal memory contribute to underestimating Alzheimer’s Disease pathology in women versus men?. J. Alzheimer’s Dis..

[28] Nebel RA (2018). Understanding the impact of sex and gender in Alzheimer’s disease: a call to action. Alzheimer’s Dementia.

[29] Sinforiani E (2010). Impact of gender differences on the outcome of alzheimer’s disease. Dement. Geriatr. Cogn. Disord..

[30] Kessler RC, McGonagle KA, Swartz M, Blazer DG, Nelson CB (1993). Sex and depression in the National Comorbidity Survey I: Lifetime prevalence, chronicity and recurrence. J Affect. Disord..

[31] Goldstein JM, Holsen L, Handa R, Tobet S (2014). Fetal hormonal programming of sex differences in depression: linking women’s mental health with sex differences in the brain across the lifespan. Front. Neurosci..

[32] Bromberger JT (2011). Major depression during and after the menopausal transition: Study of Women’s Health Across the Nation (SWAN). Psychol. Med..

[33] Cohen LS, Soares CN, Vitonis AF, Otto MW, Harlow BL (2006). Risk for new onset of depression during the menopausal transition: the harvard study of moods and cycles. Arch. Gen. Psychiatry.

[34] Ohayon, M. M., Carskadon, M. A., Guilleminault, C. & Vitiello, M. V. Meta-analysis of quantitative sleep parameters from childhood to old age in healthy individuals: developing normative sleep values across the human lifespan. *Sleep* **27**, 1255–1273 (2004). 10.1093/sleep/27.7.1255 15586779

[35] Pankratz VS (2015). Predicting the risk of mild cognitive impairment in the Mayo Clinic Study of Aging. Neurology.

[36] Ownby RL, Crocco E, Acevedo A, John V, Loewenstein D (2006). Depression and risk for Alzheimer disease: systematic review, meta-analysis, and metaregression analysis. Arch. Gen. Psychiatry.

[37] Ju YES (2017). Slow wave sleep disruption increases cerebrospinal fluid amyloid-β levels. Brain.

[38] Barnes, D. E. & Yaffe, K. The projected effect of risk factor reduction on Alzheimer’s disease prevalence. *Lancet Neurol.* **10**, 819–828 (2011). 10.1016/S1474-4422(11)70072-2 PMC3647614 21775213

[39] Jiang L, Lin H (2020). Alzheimer’s disease neuroimaging initiative & Chen, Y. Sex difference in the association of APOE4 with cerebral glucose metabolism in older adults reporting significant memory concern. Neurosci. Lett..

[40] Crawford F (2000). Gender-specific association of the angiotensin converting enzyme gene with Alzheimer’s disease. Neurosci. Lett..

[41] Li GD (2017). Female-specific effect of the BDNF gene on Alzheimer’s disease. Neurobiol. Aging.

[42] Fehér Á, Juhász A, Pákáski M, Kálmán J, Janka Z (2015). Genetic analysis of the RELN gene: gender specific association with Alzheimer’s disease. Psychiatry Res..

[43] Prokopenko D (2020). Identification of novel alzheimer’s disease loci using sex-specific family-based association analysis of whole-genome sequence data. Sci. Rep..

[44] Deming Y (2018). Sex-specific genetic predictors of Alzheimer’s disease biomarkers. Acta Neuropathol..

[45] Guo, L., Zhong, M. B., Zhang, L., Zhang, B. & Cai, D. Sex differences in Alzheimer’s Disease: insights from the multiomics landscape. *Biol. Psychiatry* **91**, 61–71 (2022). 10.1016/j.biopsych.2021.02.968 PMC8996342 33896621

[46] Ferretti, M. T. et al. Sex differences in Alzheimer disease — The gateway to precision medicine. *Nat. Rev. Neurol.* **14**, 457–469 (2018). 10.1038/s41582-018-0032-9 29985474

[47] Katsonis P, Lichtarge O (2014). A formal perturbation equation between genotype and phenotype determines the evolutionary action of protein-coding variations on fitness. Genome Res..

[48] Katsonis P, Lichtarge O (2017). Objective assessment of the evolutionary action equation for the fitness effect of missense mutations across CAGI-blinded contests. Hum. Mutat..

[49] Katsonis P, Lichtarge O (2019). CAGI5: Objective performance assessments of predictions based on the evolutionary action equation. Hum. Mutat..

[50] Koire, A. et al. A method to delineate de novo missense variants across pathways prioritizes genes linked to autism. *Sci. Transl. Med.* **13**, 594 (2021). 10.1126/scitranslmed.abc1739 PMC8916821 34011629

[51] Kim YW (2020). Harnessing the paradoxical phenotypes of APOE ɛ2 and APOE ɛ4 to identify genetic modifiers in Alzheimer’s disease. Alzheimer’s Dementia.

[52] Clarke CN (2019). Comprehensive genomic characterization of parathyroid cancer identifies novel candidate driver mutations and core pathways. J. Endocr. Soc..

[53] Ally A (2017). Comprehensive and integrative genomic characterization of hepatocellular carcinoma. Cell.

[54] Frank, E. & Witten, I. H. Generating accurate rule sets without global optimization. In *Proceeding ICML ’98 Proceedings of the Fifteenth International Conference on Machine Learning* 1-55860-556-8 (1998).

[55] Cohen, W. W. Fast Effective Rule Induction. In *Machine Learning Proceedings.*10.1016/B978-1-55860-377-6.50023-2 (1995).

[56] Ruck, D. W., Rogers, S. K., Kabrisky, M., Oxley, M. E. & Suter, B. W. Letters: The multilayer perceptron as an approximation to a bayes optimal discriminant function. *IEEE Trans. Neural Netw.*10.1109/72.80266 (1990). 10.1109/72.80266 18282850

[57] John, G. H. & Langley, P. Estimating Continuous Distributions in Bayesian Classifiers. In *Proceedings of the Eleventh Conference on Uncertainty in Artificial Intelligence.*10.48550/arXiv.1302.4964 (1995).

[58] Cessie Sle, Houwelingen JCvan (1992). Ridge estimators in logistic regression. Appl. Stat..

[59] Aha, D. W., Kibler, D. & Albert, M. K. Instance-based learning algorithms. *Mach. Learn.*10.1023/A:1022689900470 (1991).

[60] Breiman L (2001). Random forests. Mach. Learn.

[61] Quinlan, J. R. *C4.5: Programs for Machine Learning*. *Morgan Kaufmann San Mateo California* 273. 10.1001/jama.1995.03520250075037 (1992).

[62] Freund, Y. & Schapire, R. R. E. Experiments with a new boosting algorithm. In *ICML'96: Proceedings of the Thirteenth International Conference on International Conference on Machine Learning*. 148–156 (1996).

[63] Matthews BW (1975). Comparison of the predicted and observed secondary structure of T4 phage lysozyme. BBA - Protein Structure.

[64] Lisewski AM, Lichtarge O (2010). Untangling complex networks: risk minimization in financial markets through accessible spin glass ground states. Phys. A: Stat. Mech. Appl..

[65] Venner E (2010). Accurate protein structure annotation through competitive diffusion of enzymatic functions over a network of local evolutionary similarities. PLoS One.

[66] Lisewski, A. M. et al. Supergenomic network compression and the discovery of exp1 as a glutathione transferase inhibited by artesunate. *Cell* **158**, 916–928 (2014). 10.1016/j.cell.2014.07.011 PMC4167585 25126794

[67] Pham, M. & Lichtarge, O. Graph-based information diffusion method for prioritizing functionally related genes in protein-protein interaction networks. *Pac Symp Biocomput* **25**, 439–450 (2020). PMC7043368 31797617

[68] Cowen, L., Ideker, T., Raphael, B. J. & Sharan, R. Network propagation: a universal amplifier of genetic associations. *Nat. Rev. Genet.* **18**, 551–562 (2017). 10.1038/nrg.2017.38 28607512

[69] Alako BTF (2005). CoPub Mapper: Mining MEDLINE based on search term co-publication. BMC Bioinform..

[70] Pham M, Wilson S, Govindarajan H, Lin CH, Lichtarge O (2020). Discovery of disease- And drug-specific pathways through community structures of a literature network. Bioinformatics.

[71] Wilson, S. J. et al. Automated literature mining and hypothesis generation through a network of Medical Subject Headings. *bioRxiv*. Preprint at 10.1101/403667 (2018).

[72] Allen M (2016). Human whole genome genotype and transcriptome data for Alzheimer’s and other neurodegenerative diseases. Sci. Data.

[73] de Jager PL (2018). Data descriptor: a multi-omic atlas of the human frontal cortex for aging and Alzheimer’s disease research. Sci. Data.

[74] Mostafavi S (2018). A molecular network of the aging human brain provides insights into the pathology and cognitive decline of Alzheimer’s disease. Nat. Neurosci..

[75] Wan YW (2020). Meta-analysis of the Alzheimer’s Disease human brain transcriptome and functional dissection in mouse models. Cell Rep..

[76] Logsdon, B. A. et al. Meta-analysis of the human brain transcriptome identifies heterogeneity across human AD coexpression modules robust to sample collection and methodological approach. *bioRxiv* 510420. 10.1101/510420 (2019).

[77] Hodes, R. J. & Buckholtz, N. Accelerating Medicines Partnership: Alzheimer’s Disease (AMP-AD) Knowledge Portal Aids Alzheimer’s Drug Discovery through Open Data Sharing. *Expert Opin Ther Targets* **20**. 389–391 (2016). 10.1517/14728222.2016.1135132 26853544

[78] Preuss C (2020). A novel systems biology approach to evaluate mouse models of late-onset Alzheimer’s disease. Mol. Neurodegener.

[79] Zhang Y (2014). An RNA-sequencing transcriptome and splicing database of glia, neurons, and vascular cells of the cerebral cortex. J. Neurosci..

[80] Zhang Y (2016). Purification and characterization of progenitor and mature human astrocytes reveals transcriptional and functional differences with mouse. Neuron.

[81] Zhu J (2013). Integrated systems approach identifies genetic nodes and networks in late-onset Alzheimer’s Disease. Cell.

[82] Lasagna-Reeves CA (2016). Reduction of Nuak1 decreases Tau and reverses phenotypes in a Tauopathy mouse model. Neuron.

[83] Chouhan AK (2016). Uncoupling neuronal death and dysfunction in Drosophila models of neurodegenerative disease. Acta Neuropathol. Commun..

[84] Onur, T. S. et al. Downregulation of glial genes involved in synaptic function mitigates huntington’s disease pathogenesis. *Elife* **10**, e64564 (2021). 10.7554/eLife.64564 PMC8149125 33871358

[85] Rousseaux MWC (2018). A druggable genome screen identifies modifiers of α-synuclein levels via a tiered cross-species validation approach. J. Neurosci..

[86] Park J (2013). RAS-MAPK-MSK1 pathway modulates ataxin 1 protein levels and toxicity in SCA1. Nature.

[87] Domingos, P. & Hulten, G. Mining high-speed data streams. In *Proceeding of the Sixth ACM SIGKDD International Conference on Knowledge Discovery and Data Mining*10.1145/347090.347107 (2000).

[88] Lee S (2012). Optimal unified approach for rare-variant association testing with application to small-sample case-control whole-exome sequencing studies. Am. J. Hum. Genet..

[89] KENDALL MGA (1938). New measure of rank correlation. Biometrika.

[90] Steinberg S (2015). Loss-of-function variants in ABCA7 confer risk of Alzheimer’s disease. Nat. Genet..

[91] Bellenguez C (2017). Contribution to Alzheimer’s disease risk of rare variants in TREM2, SORL1, and ABCA7 in 1779 cases and 1273 controls. Neurobiol. Aging.

[92] Chen, Y. C. et al. Performance metrics for selecting single nucleotide polymorphisms in late-onset Alzheimer’s Disease. *Sci. Rep.* **6**, 36155 (2016). 10.1038/srep36155 PMC5090242 27805002

[93] Harold D (2009). Genome-wide association study identifies variants at CLU and PICALM associated with Alzheimer’s disease. Nat. Genet..

[94] Lambert JC (2009). Genome-wide association study identifies variants at CLU and CR1 associated with Alzheimer’s disease. Nat. Genet..

[95] Lin YL (2012). Genetic polymorphisms of clusterin gene are associated with a decreased risk of Alzheimer’s disease. Eur. J. Epidemiol..

[96] Fattahi, M. J. & Mirshafiey, A. Positive and negative effects of prostaglandins in Alzheimer’s disease. *Psychiatry Clin. Neurosci.* **68**, 50–60 (2014). 10.1111/pcn.12092 23992456

[97] Bazan NG, Colangelo V, Lukiw WJ (2002). Prostaglandins and other lipid mediators in Alzheimer’s disease. Prostaglandins Other Lipid Mediat..

[98] Liang X (2005). Deletion of the prostaglandin E2 EP2 receptor reduces oxidative damage and amyloid burden in a model of Alzheimer’s disease. J. Neurosci..

[99] Johansson JU (2015). Prostaglandin signaling suppresses beneficial microglial function in Alzheimer’s disease models. J. Clin. Investig..

[100] Woodling NS (2014). Suppression of Alzheimer-associated inflammation by microglial prostaglandin-E2 EP4 receptor signaling. J. Neurosci..

[101] Wood H (2012). Prostaglandin E2 signalling is implicated in inflammation early in the Alzheimer disease course. Nat. Rev. Neurol..

[102] Grill M, Heinemann A, Hoefler G, Peskar BA, Schuligoi R (2008). Effect of endotoxin treatment on the expression and localization of spinal cyclooxygenase, prostaglandin synthases, and PGD2 receptors. J. Neurochem..

[103] Nakajo A (2016). EHBP1L1 coordinates Rab8 and Bin1 to regulate apical-directed transport in polarized epithelial cells. J. Cell Biol..

[104] Gate D (2020). Clonally expanded CD8 T cells patrol the cerebrospinal fluid in Alzheimer’s disease. Nature.

[105] Huang F (2019). CDT2-controlled cell cycle reentry regulates the pathogenesis of Alzheimer’s disease. Alzheimer’s Dementia.

[106] Paranjpe, M. D. et al. Sex-specific cross tissue meta-analysis identifies immune dysregulation in women with Alzheimer’s Disease, sex-specific cross tissue meta-analysis identifies immune dysregulation in women with Alzheimer’s Disease. *Front Aging Neurosci* **13**, 735611 (2020). 10.3389/fnagi.2021.735611 PMC8515049 34658838

[107] Shulman JM (2013). Genetic susceptibility for Alzheimer disease neuritic plaque pathology. JAMA Neurol..

[108] Escott-Price V (2014). Gene-wide analysis detects two new susceptibility genes for Alzheimer’s disease. PLoS One.

[109] Stone JG (2011). The cell cycle regulator phosphorylated retinoblastoma protein is associated with tau pathology in several tauopathies. J. Neuropathol. Exp. Neurol..

[110] Silva ART (2014). Repair of oxidative DNA damage, cell-cycle regulation and neuronal death may influence the clinical manifestation of Alzheimer’s disease. PLoS One.

[111] Delobel, P., Lavenir, I., Ghetti, B., Holzer, M. & Goedert, M. Cell-cycle markers in a transgenic mouse model of human tauopathy: increased levels of cyclin-dependent kinase inhibitors p21Cip1 and p27Kip1. *Am. J. Pathol.* **168**, 878–887 (2006). 10.2353/ajpath.2006.050540 PMC1606514 16507903

[112] McShea A, Harris PLR, Webster KR, Wahl AF, Smith MA (1997). Abnormal expression of the cell cycle regulators P16 and CDK4 in Alzheimer’s disease. Am. J. Pathol..

[113] Nagy Z, Esiri MM, Cato AM, Smith AD (1997). Cell cycle markers in the hippocampus in Alzheimer’s disease. Acta Neuropathol..

[114] Markesbery WR, Carney JM (1999). Oxidative alterations in Alzheimer’s disease. in. Brain Pathol..

[115] Sonoda Y (2010). Accumulation of tumor-suppressor PTEN in Alzheimer neurofibrillary tangles. Neurosci. Lett..

[116] Mano T (2017). Neuron-specific methylome analysis reveals epigenetic regulation and tau-related dysfunction of BRCA1 in Alzheimer’s disease. Proc. Natl Acad. Sci. USA.

[117] Evans TA (2007). BRCA1 may modulate neuronal cell cycle re-entry in Alzheimer disease. Int. J. Med. Sci..

[118] Nakanishi A, Minami A, Kitagishi Y, Ogura Y, Matsuda S (2015). BRCA1 and p53 tumor suppressor molecules in Alzheimer’S disease. Int. J. Mol. Sci..

[119] Suberbielle E (2015). DNA repair factor BRCA1 depletion occurs in Alzheimer brains and impairs cognitive function in mice. Nat. Commun..

[120] Ojelade SA (2019). cindr, the Drosophila Homolog of the CD2AP Alzheimer’s disease risk gene, is required for synaptic transmission and proteostasis. Cell Rep..

[121] Ratnu, V. S., Emami, M. R. & Bredy, T. W. Genetic and epigenetic factors underlying sex differences in the regulation of gene expression in the brain. *J. Neurosci. Res.* **95**, 301–310 (2017). 10.1002/jnr.23886 PMC5120607 27870402

[122] Liang X (2009). Genomic convergence to identify candidate genes for Alzheimer disease on chromosome 10. Hum. Mutat..

[123] Sims R (2017). Rare coding variants in PLCG2, ABI3, and TREM2 implicate microglial-mediated innate immunity in Alzheimer’s disease. Nat. Genet..

[124] Conway OJ (2018). ABI3 and PLCG2 missense variants as risk factors for neurodegenerative diseases in Caucasians and African Americans. Mol. Neurodegener.

[125] Xu Z, Wu C, Pan W (2017). Imaging-wide association study: integrating imaging endophenotypes in GWAS. Neuroimage.

[126] Liao X (2020). Identification of Alzheimer’s disease–associated rare coding variants in the ECE2 gene. JCI Insight.

[127] Turner RS (2020). Nilotinib effects on safety, tolerability, and biomarkers in Alzheimer’s disease. Ann. Neurol..

[128] Estrada LD (2016). Reduction of blood amyloid-β oligomers in Alzheimer’s disease transgenic mice by c-Abl kinase inhibition. J. Alzheimer’s Dis..

[129] Huang L (2016). Sunitinib, a clinically used anticancer drug, is a potent AChE inhibitor and attenuates cognitive impairments in mice. ACS Chem. Neurosci..

[130] Han KM (2020). Regorafenib regulates AD pathology, neuroinflammation, and dendritic spinogenesis in cells and a mouse model of AD. Cells.

[131] Logue MW (2019). Use of an Alzheimer’s disease polygenic risk score to identify mild cognitive impairment in adults in their 50s. Mol Psychiatry.

[132] Mormino EC (2016). Polygenic risk of Alzheimer disease is associated with early- and late-life processes. Neurology.

[133] Tosto G (2017). Polygenic risk scores in familial Alzheimer disease. Neurology.

[134] Escott-Price V (2015). Common polygenic variation enhances risk prediction for Alzheimer’s disease. Brain.

[135] Escott-Price V, Myers AJ, Huentelman M, Hardy J (2017). Polygenic risk score analysis of pathologically confirmed Alzheimer disease. Ann. Neurol..

[136] Chuang LC, Kuo PH (2017). Building a genetic risk model for bipolar disorder from genome-wide association data with random forest algorithm. Sci. Rep..

[137] Kruppa J, Ziegler A, König IR (2012). Risk estimation and risk prediction using machine-learning methods. Hum. Genet..

[138] Griswold AJ (2021). Increased APOE ε4 expression is associated with the difference in Alzheimer’s disease risk from diverse ancestral backgrounds. Alzheimer’s Dementia.

[139] Matthews KA (2019). Racial and ethnic estimates of Alzheimer’s disease and related dementias in the United States (2015–2060) in adults aged ≥65 years. Alzheimer’s Dementia.

[140] Rajabli F (2022). A locus at 19q13.31 significantly reduces the ApoE ε4 risk for Alzheimer’s Disease in African Ancestry. PLoS Genet..

[141] Fenoglio, C., Scarpini, E., Serpente, M. & Galimberti, D. Role of Genetics and Epigenetics in the Pathogenesis of Alzheimer’s Disease and Frontotemporal Dementia. *J. Alzheimer’s Dis.* **62**, 913–932 (2018). 10.3233/JAD-170702 PMC5870004 29562532

[142] Wigginton, J. E., Cutler, D. J. & Abecasis, G. R. A note on exact tests of Hardy-Weinberg equilibrium, Am J Hum Genet. **76**, 887–893 (2005). 10.1086/429864 PMC1199378 15789306

[143] Wang K, Li M, Hakonarson H (2010). ANNOVAR: Functional annotation of genetic variants from high-throughput sequencing data. Nucleic Acids Res..

[144] Danecek P (2021). Twelve years of SAMtools and BCFtools. Gigascience.

[145] Purcell S (2007). PLINK: a tool set for whole-genome association and population-based linkage analyses. Am. J. Hum. Genet..

[146] Manichaikul A (2010). Robust relationship inference in genome-wide association studies. Bioinformatics.

[147] Patterson N, Price AL, Reich D (2006). Population structure and eigenanalysis. PLoS Genet..

[148] Guerreiro R (2013). *TREM2* variants in Alzheimer’s disease. N. Engl. J. Med..

[149] Jonsson T (2013). Variant of *TREM2* associated with the risk of Alzheimer’s disease. N. Engl. J. Med..

[150] Lichtarge O, Bourne HR, Cohen FE (1996). An evolutionary trace method defines binding surfaces common to protein families. J. Mol. Biol..

[151] Hansen LK, Salamon P (1990). Neural network ensembles. IEEE Trans. Pattern Anal. Mach. Intell..

[152] Opitz D, Maclin R (1999). Popular ensemble methods: an empirical study. J. Artificial Intelligence Res..

[153] Dietterich, T. G. Ensemble methods in machine learning. In: Multiple Classifier Systems. MCS 2000. Lecture Notes in Computer Science, vol 1857. Springer, Berlin, Heidelberg. 10.1007/3-540-45014-9_1.

[154] McKenzie AT (2018). Brain cell type specific gene expression and co-expression network architectures. Sci. Rep..

[155] Gu C (2013). HPLC and UPLC-MS detection of 5-HMF from rabbit ncurolymph after treated with Cornus officinalis. Smoothing Spline: ANOVA Models.

[156] Wang M, Zhao Y, Zhang B (2015). Efficient test and visualization of multi-set intersections. Sci. Rep..

