# Supplementary material for: Functional variants identify sex-specific genes and pathways in Alzheimer’s Disease
Source: Nat Commun. 2023 May 13;14:2765. doi: 10.1038/s41467-023-38374-z (PMC10183026; doi:10.1038/s41467-023-38374-z)
Supplement: Supplementary file 1 — Supplementary Information [file 41467_2023_38374_MOESM1_ESM.pdf]

Supplementary Figure 1. Heatmap of dysregulated EAML genes

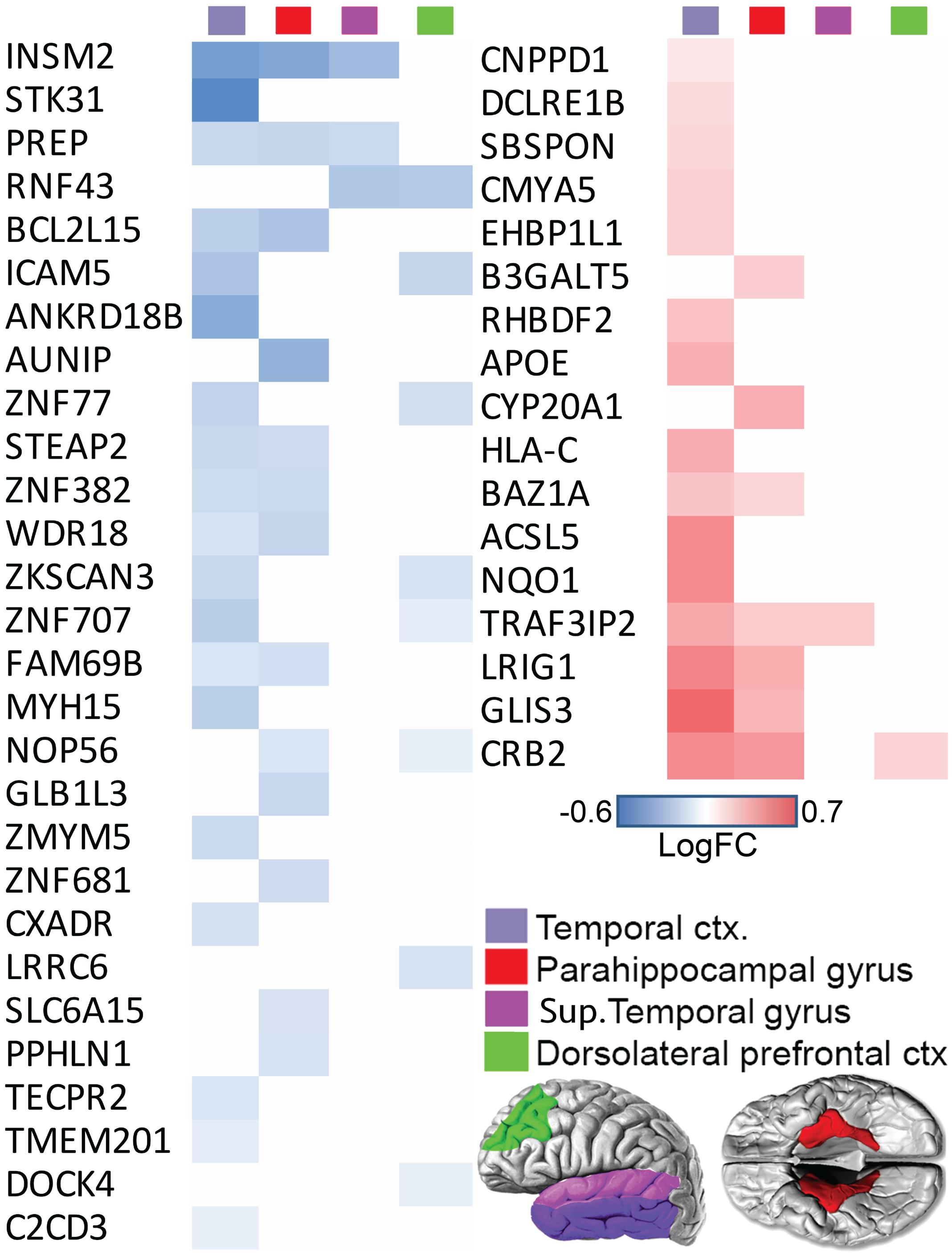

The heat map showing all the EAML candidates dysregulated in at least one brain region in AD brains compared to controls.

Supplementary Figure 2. Functional integration of EAML hits with AD-specific brain transcriptomic data

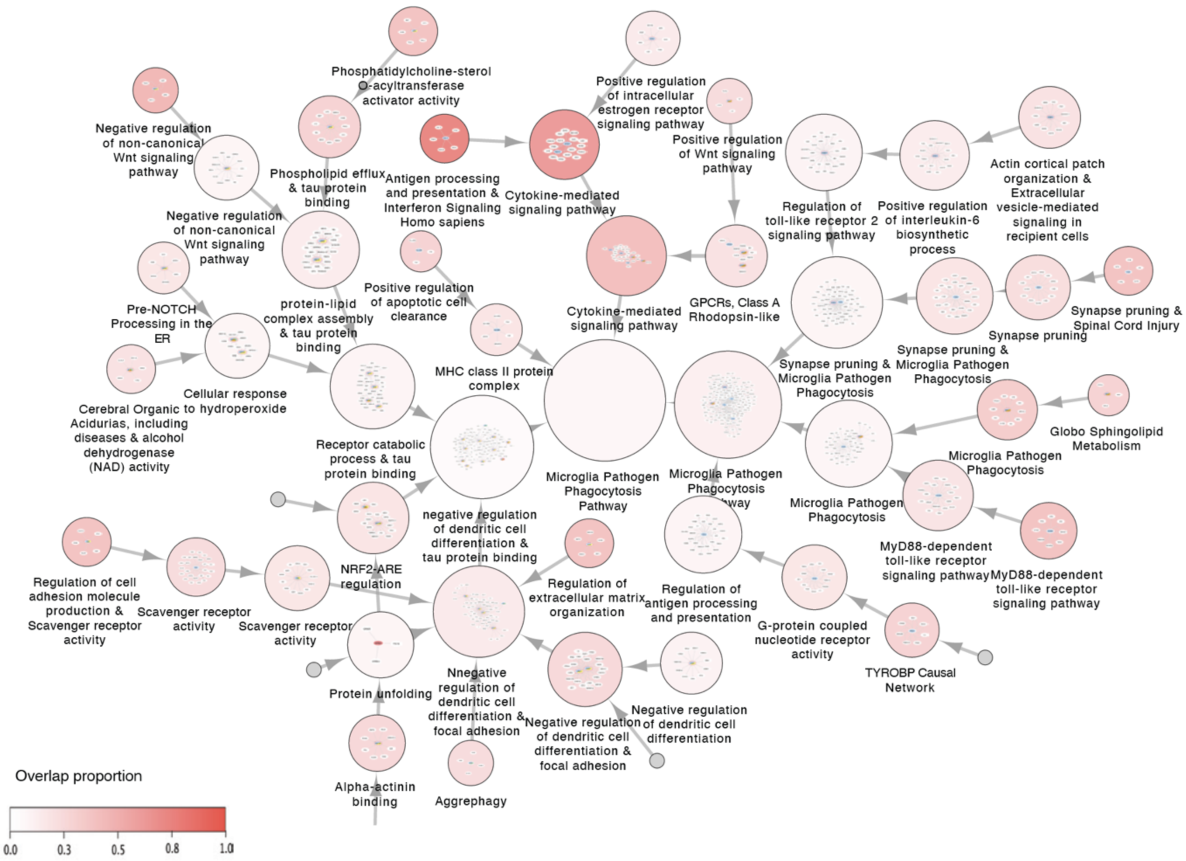

Mapping of EAML candidate genes into AD-specific coexpression networks. Modules were built using the HiDef-Louvain algorithm tool in the Community Detection extension applied to the coexpression network and functional enrichment analysis was performed using the community detection module in Cytoscape. Each module's color indicates the proportion of overlap between the genes in the module and the genes classified as members of the functional pathway in the database.

Supplementary Figure 3A. EAML candidates that modulate Tau-induced neuronal dysfunction in vivo

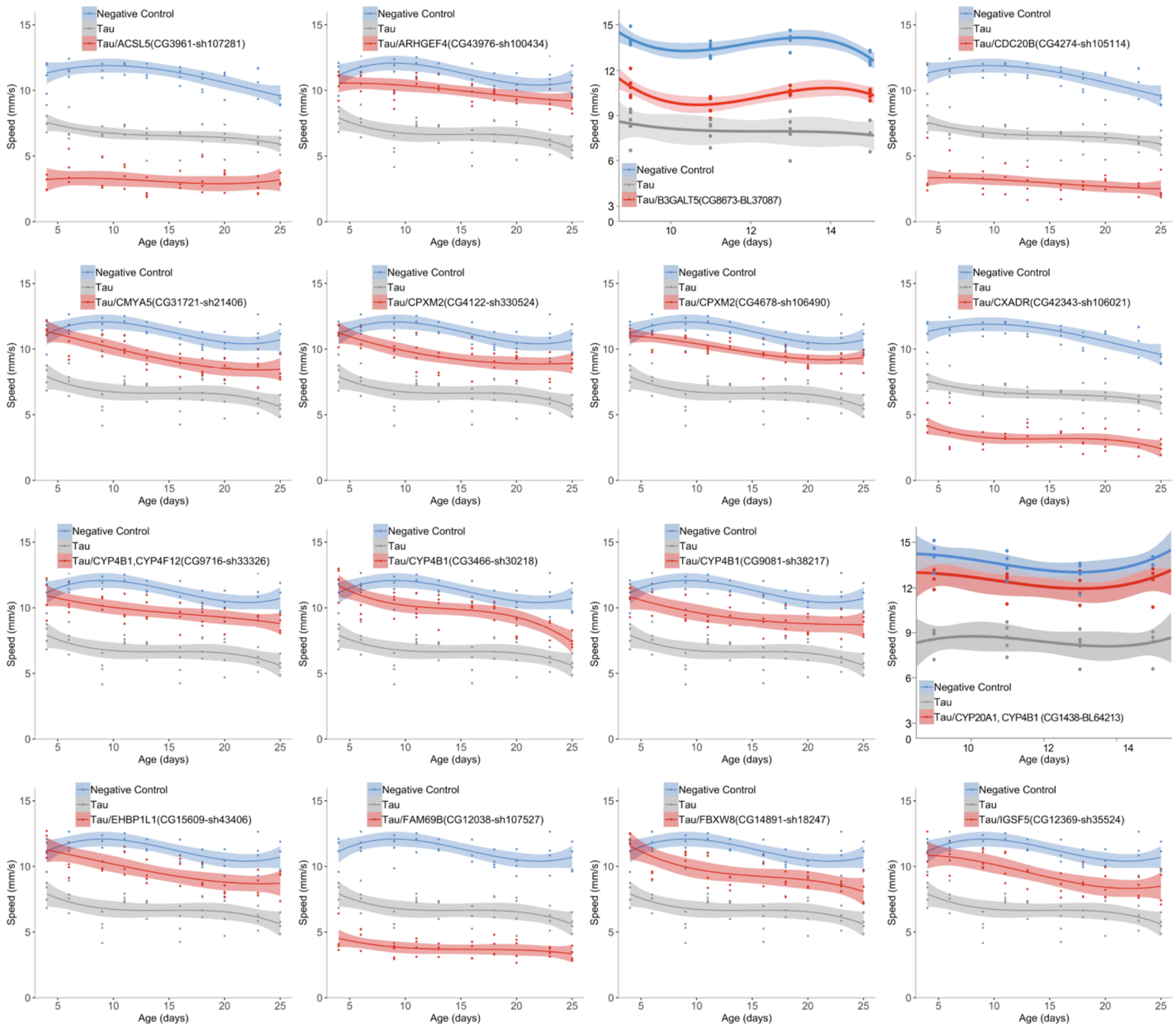

Supplementary Figure 3A. EAML candidates that modulate Tau-induced neuronal dysfunction in vivo (continued)

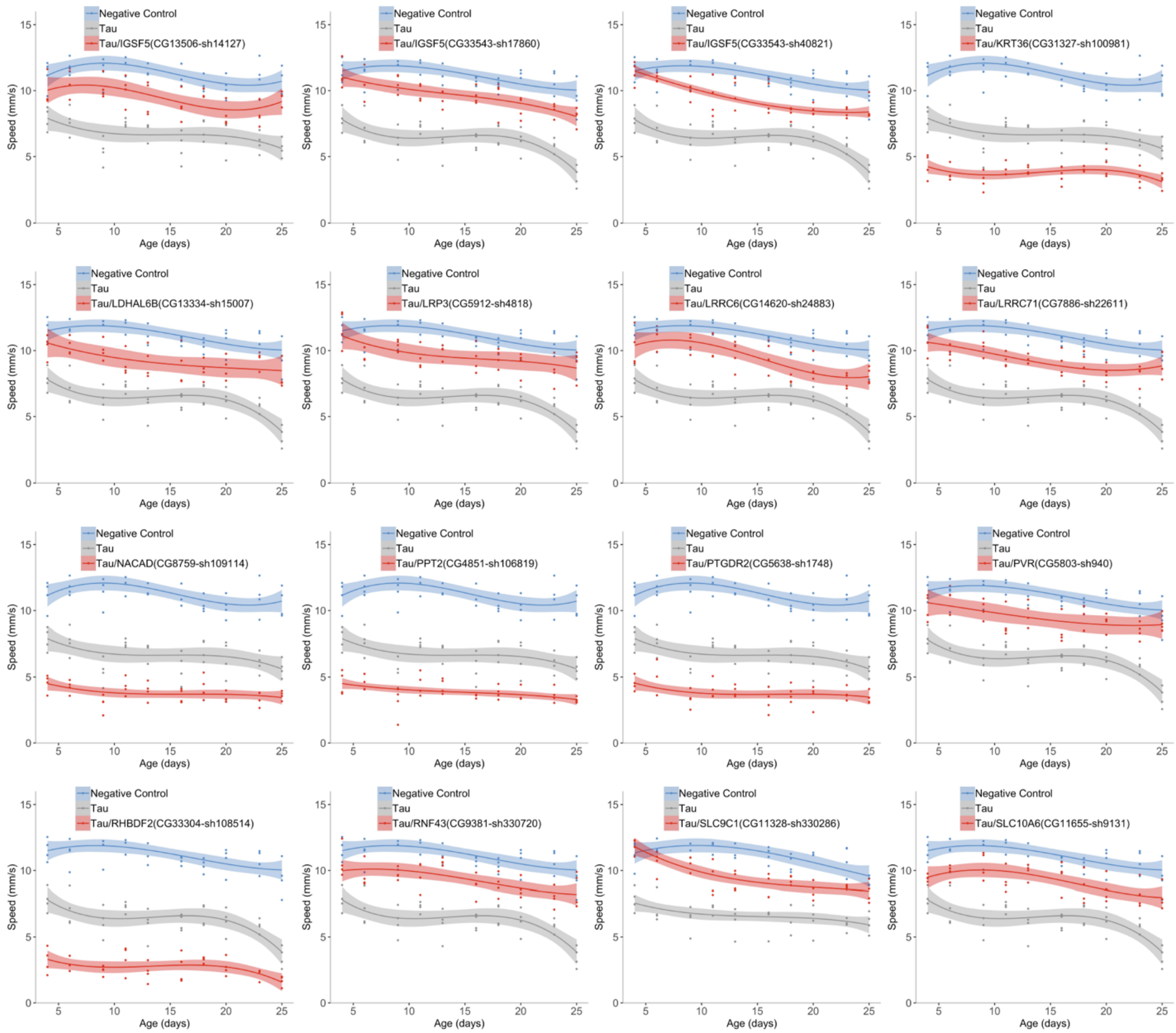

Supplementary Figure 3A. EAML candidates that modulate Tau-induced neuronal dysfunction in vivo (continued)

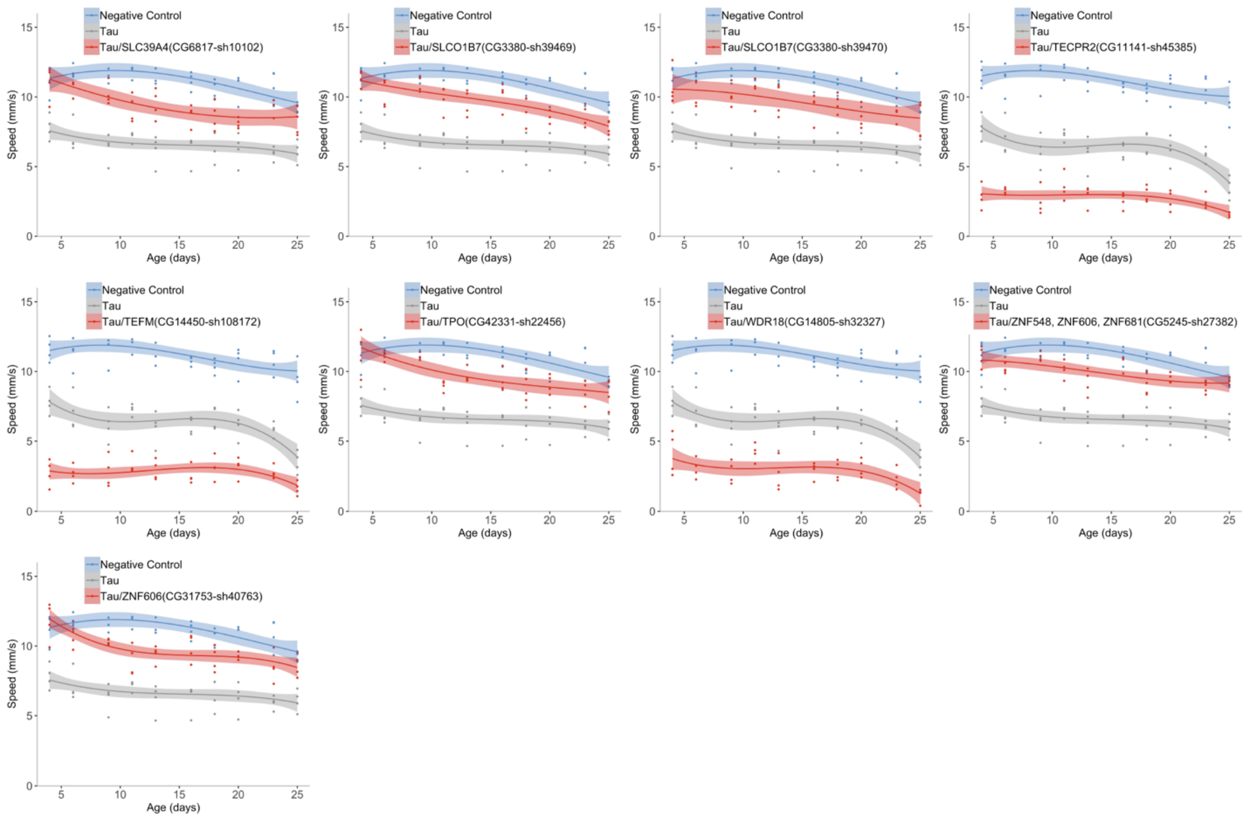

Supplementary Figure 3B. EAML candidates that modulate 42-induced neuronal dysfunction in vivo

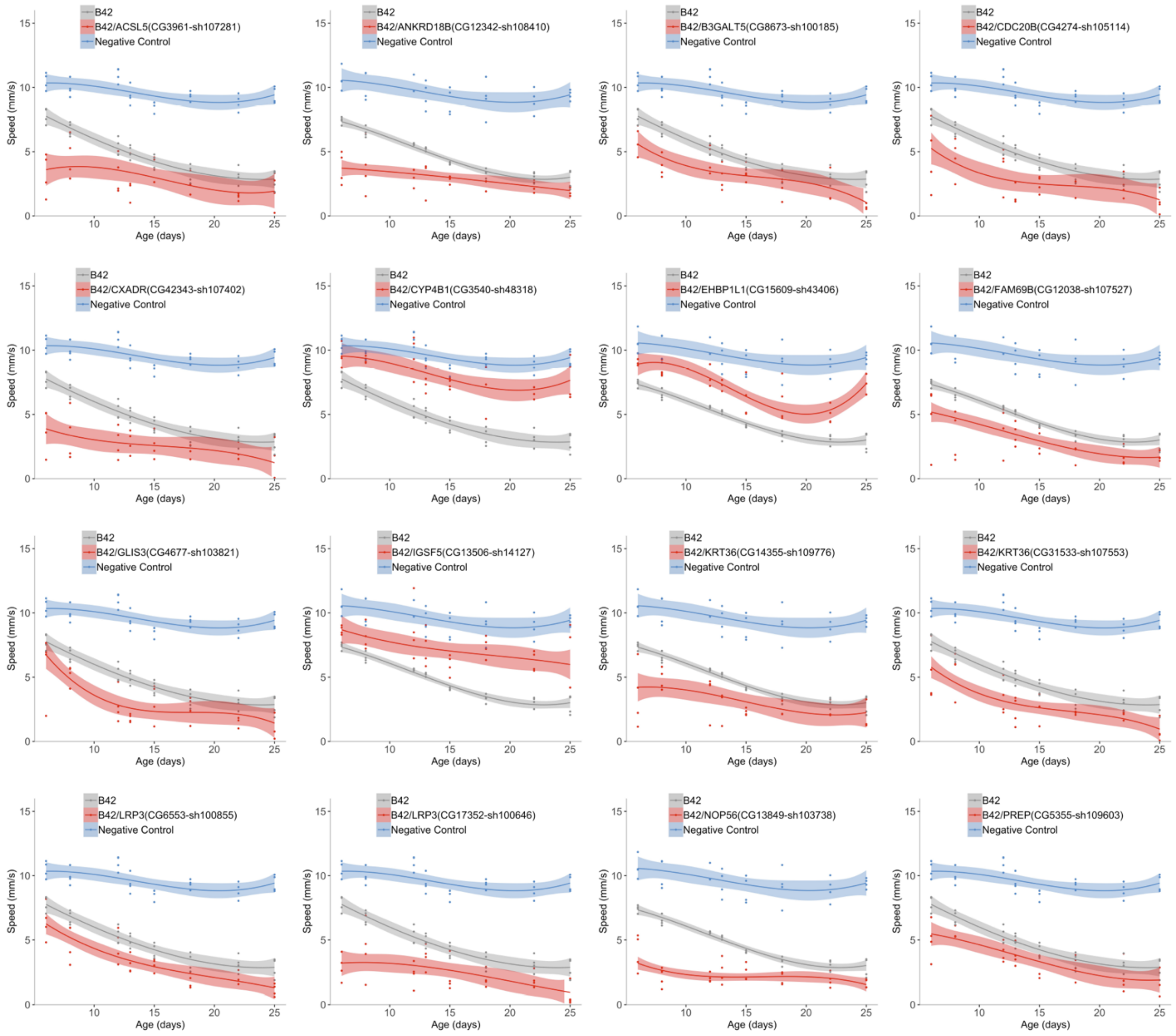

Supplementary Figure 3B. EAML candidates that modulate 42-induced neuronal dysfunction in vivo (continued)

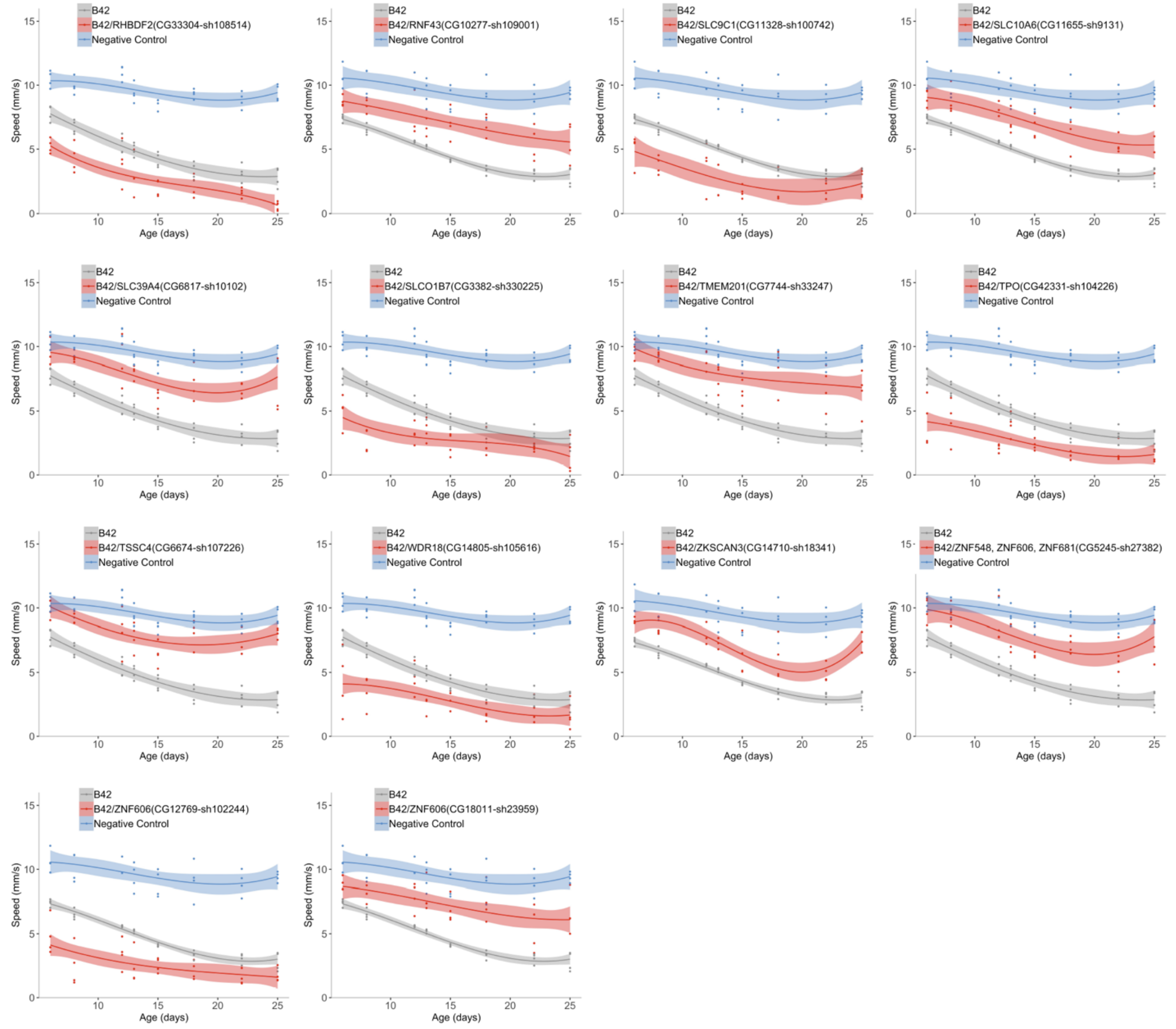

Specific graphs representing speed as a function of age for the indicated allele or shRNA. Blue corresponds to negative controls expressing a non-targeting hp-RNA. Black shows the performance of tau/non-targeting (A) or ß42/non-targeting hp-RNA (B) as a function of age. Red shows the performance of animals carrying the allele/ shRNA indicated on top and either expressing tau (A) or ß42(B) paneuronally. Each dataset is represented as a spline regression and the shaded area represents the confidence intervals. All charts shown had a statistically significant effect ($p<0.05$) when comparing them using ANOVA applied to non-linear mixed model regressions with splines. Specific p-values for each graph are indicated in Supplementary Data2. Each regression was generated using data from 4 independent replicates and each replicate had 10 age matched females collected within a 24hr period.

Supplementary Figure 4. Down-sampling Analyses - Kendall Tau ranking test

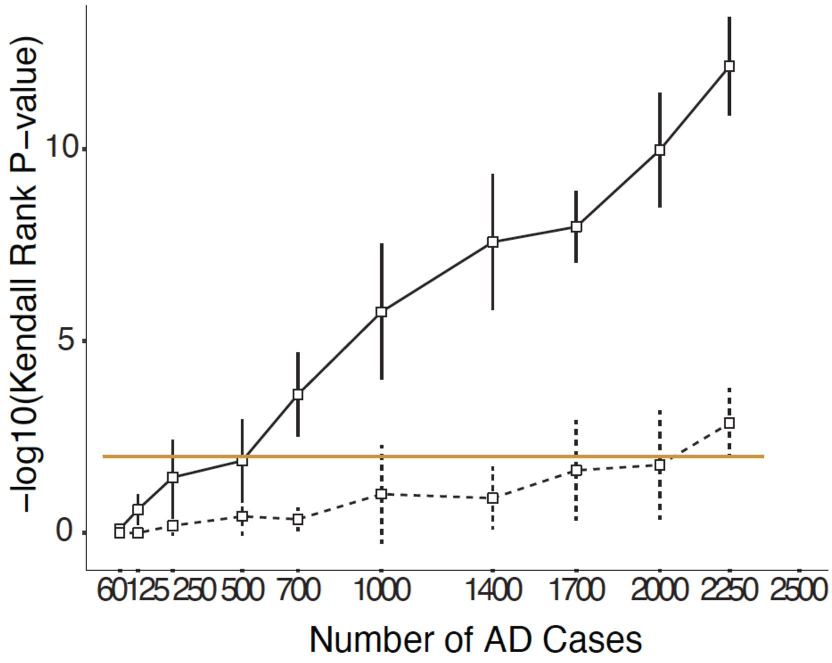

X indicates the number of randomly selected samples of AD cases; an equal number of control samples were also randomly selected. Y indicates Kendall-Tau ranking test P-values comparing the top rankings of 50 genes from each iterated experiment to the rankings of top 50 genes from full cohort, solid line indicates EAML while dot line indicates SKAT-O. The error bar represents standard error for the mean of Kendall Tau ranking p-values (one-sided).

Supplementary Figure 5. Integration of Male and Female specific EAML candidates with expression network dysregulated in AD

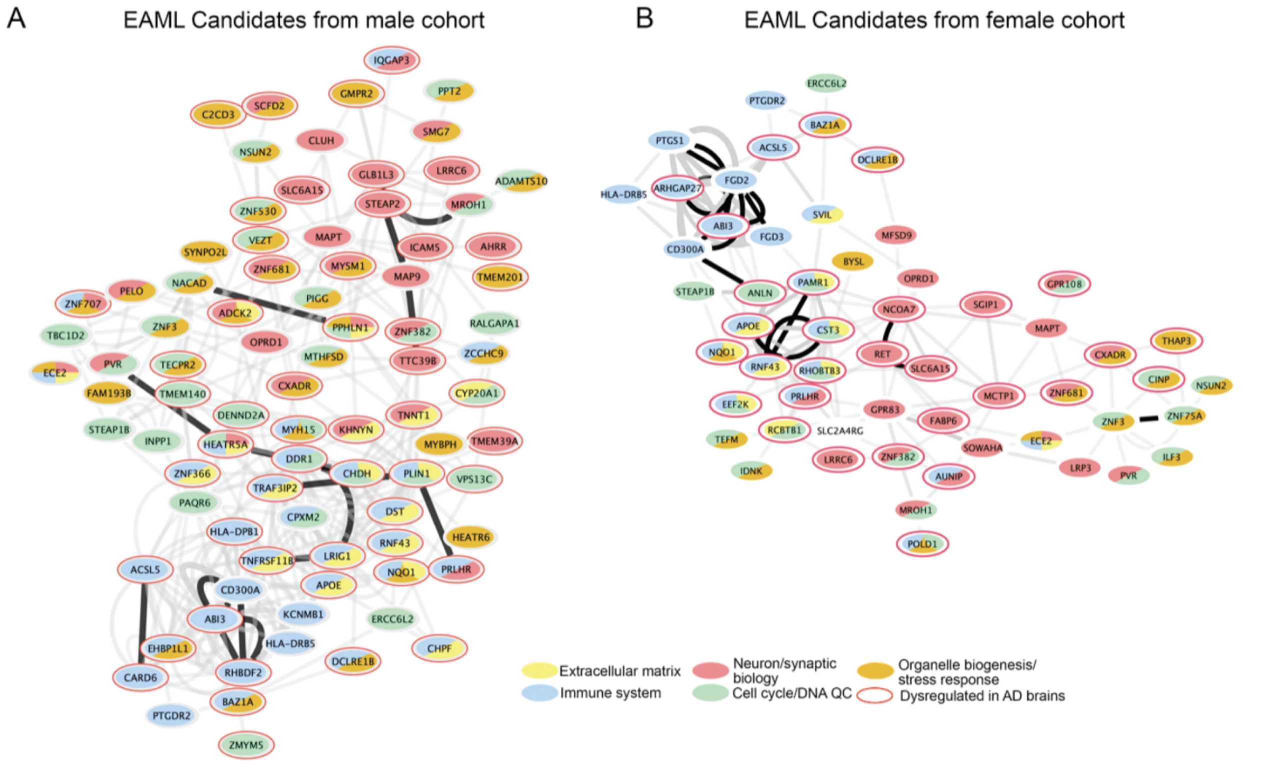

(A) Male specific and (B) female specific EAML candidates mapped into AD consensus modules network (organic distribution) based on gene co-expression analysis.[1,2,3] The main function enriched in each module [1,2,3] is indicated. Darker, thicker edges connect genes that are more highly correlated. Genes with a red ring are dysregulated in at least one brain region in AD vs. control.

Supplementary Table 1. 25 AD GWAS genes

25 GWAS genes
ABCA7
ACE
ADAM10
ADAMTS1
APOE
BIN1
CASS4
CD2AP
CLU
CR1
ECHDC3
EPHA1
FERMT2
HLA -DRB1
INPP5D
IQCK
MS4A2
NYAP1
PICALM
PTK2B
SLC24A4
SORL1
SPI1
TREM2
WWOX

Supplementary References

1. Wan, Y. W. *et al.* Meta-Analysis of the Alzheimer's Disease Human Brain Transcriptome and Functional Dissection in Mouse Models. *Cell Rep* **32**, (2020).
2. Logsdon, B. A. *et al.* Meta-analysis of the human brain transcriptome identifies heterogeneity across human AD coexpression modules robust to sample collection and methodological approach. *bioRxiv* 510420 (2019) doi:10.1101/510420.
3. Preuss, C. *et al.* A novel systems biology approach to evaluate mouse models of late-onset Alzheimer's disease. *Mol Neurodegener* **15**, (2020).
