# Supplementary material for: Functional variants identify sex-specific genes and pathways in Alzheimer’s Disease
Source: Nat Commun. 2023 May 13;14:2765. doi: 10.1038/s41467-023-38374-z (PMC10183026; doi:10.1038/s41467-023-38374-z)
Supplement: Supplementary file 2 — Description of Additional Supplementary Files [file 41467_2023_38374_MOESM2_ESM.pdf]

**Description of Additional Supplementary Files**

File Name: Supplementary  Data 1
Description: top EAML candidate genes of Full, Male, and Female cohort

File Name: Supplementary Data 2
Description: List of Tau and β42 suppressors and enhancers in the Drosophila screen. Includes, homolog, line tested and source and p values for non linear random mixed effects model ANOVA comparissons using splines as described in materials and methods.

File Name: Supplementary Data 3
Description: Recurrent genes over downsampling analyses
