# Supplementary material for: Functional variants identify sex-specific genes and pathways in Alzheimer’s Disease
Source: Nat Commun. 2023 May 13;14:2765. doi: 10.1038/s41467-023-38374-z (PMC10183026; doi:10.1038/s41467-023-38374-z)
Supplement: Supplementary file 4 — Reporting Summary [file 41467_2023_38374_MOESM4_ESM.pdf]

nature portfolio

Corresponding author(s): Olivier Lichtarge, MD, PhD
Cullen Foundation Professor, Department of Molecular and Human Genetics,
Baylor College of Medicine

Last updated by author(s): Apr 21, 2023

Reporting Summary

Nature Portfolio wishes to improve the reproducibility of the work that we publish. This form provides structure for consistency and transparency in reporting. For further information on Nature Portfolio policies, see our Editorial Policies and the Editorial Policy Checklist.

Statistics

For all statistical analyses, confirm that the following items are present in the figure legend, table legend, main text, or Methods section.

n/a	Confirmed	
☐	☒	The exact sample size (*n*) for each experimental group/condition, given as a discrete number and unit of measurement
☐	☒	A statement on whether measurements were taken from distinct samples or whether the same sample was measured repeatedly
☐	☒	The statistical test(s) used AND whether they are one- or two-sided<br>*Only common tests should be described solely by name; describe more complex techniques in the Methods section.*
☐	☒	A description of all covariates tested
☐	☒	A description of any assumptions or corrections, such as tests of normality and adjustment for multiple comparisons
☐	☒	A full description of the statistical parameters including central tendency (e.g. means) or other basic estimates (e.g. regression coefficient) AND variation (e.g. standard deviation) or associated estimates of uncertainty (e.g. confidence intervals)
☐	☒	For null hypothesis testing, the test statistic (e.g. *F*, *t*, *r*) with confidence intervals, effect sizes, degrees of freedom and *P* value noted<br>*Give P values as exact values whenever suitable.*
☒	☐	For Bayesian analysis, information on the choice of priors and Markov chain Monte Carlo settings
☒	☐	For hierarchical and complex designs, identification of the appropriate level for tests and full reporting of outcomes
☒	☐	Estimates of effect sizes (e.g. Cohen's *d*, Pearson's *r*), indicating how they were calculated

Our web collection on statistics for biologists contains articles on many of the points above.

Software and code

Policy information about availability of computer code

Data collection	dbGaP phs000572.v7.p4,
Data analysis	bcftools 1.9, PLINK1.9 1.07, KING 2.1.6, EPACTS 3.3.0, Eigenstrat 7.2.1, R 3.3.3, Python 3.7, Weka 3.8.0, The code for the motor performace analysis is available upon request from the Botas Laboratory.

For manuscripts utilizing custom algorithms or software that are central to the research but not yet described in published literature, software must be made available to editors and reviewers. We strongly encourage code deposition in a community repository (e.g. GitHub). See the Nature Portfolio guidelines for submitting code & software for further information.

Data

Policy information about availability of data

All manuscripts must include a data availability statement. This statement should provide the following information, where applicable:

- Accession codes, unique identifiers, or web links for publicly available datasets
- A description of any restrictions on data availability
- For clinical datasets or third party data, please ensure that the statement adheres to our policy

WES: dbGaP phs000572.v7.p4; AMP-AD: syn8484987, syn8466812, syn8456629; EA score: http://eaction.lichtargelab.org/, EAML code: https://github.com/LichtargeLab/EAML

Human research participants

Policy information about studies involving human research participants and Sex and Gender in Research.

Reporting on sex and gender	We used biological attribute - Sex. The sex information (male or female) of samples is obtained from the provided phenotype information with the dbGaP data.
Population characteristics	We used Age and Genotype information.
Recruitment	NA
Ethics oversight	This study was approved by the Baylor College of Medicine IRB with the study protocol (H-37394).

Note that full information on the approval of the study protocol must also be provided in the manuscript.

Field-specific reporting

Please select the one below that is the best fit for your research. If you are not sure, read the appropriate sections before making your selection.

☒ Life sciences ☐ Behavioural & social sciences ☐ Ecological, evolutionary & environmental sciences

For a reference copy of the document with all sections, see nature.com/documents/nr-reporting-summary-flat.pdf

Life sciences study design

All studies must disclose on these points even when the disclosure is negative.

Sample size	We followed the sample size provided by the dbGaP data-set (phs000572.v7.p4).
Data exclusions	We excluded non-European white samples due to the limited number of samples.
Replication	Due to the lack of independent AD WES/WGS cohort(s), we could not analyze a replication.
Randomization	We applied randomizations in the downsampling experiment, we randomly selected cases and controls by repeating 10 iterations.
Blinding	Experimental testing and data acquisition was performed using a robot assisted behavioral platform that tests all the specimens in identical conditions and is 100% blind to experimental groups.

Reporting for specific materials, systems and methods

We require information from authors about some types of materials, experimental systems and methods used in many studies. Here, indicate whether each material, system or method listed is relevant to your study. If you are not sure if a list item applies to your research, read the appropriate section before selecting a response.

Materials & experimental systems

n/a	Involved in the study	
☒	☐	Antibodies
☒	☐	Eukaryotic cell lines
☒	☐	Palaeontology and archaeology
☐	☒	Animals and other organisms
☒	☐	Clinical data
☒	☐	Dual use research of concern

Methods

n/a	Involved in the study	
☒	☐	ChIP-seq
☒	☐	Flow cytometry
☒	☐	MRI-based neuroimaging

Animals and other research organisms

Policy information about studies involving animals; ARRIVE guidelines recommended for reporting animal research, and Sex and Gender in Research

Laboratory animals	The study was conducted using female Drosophila melanogaster. This model is not considered a laboratory animal by the Animal Welfare Act (AWA).

Wild animals	No wild animals used in this study.
Reporting on sex	The experimental testing was performed on female Drosophila melanogaster.
Field-collected samples	No field-collected samples used in this study.
Ethics oversight	Since Drosophila is not considered a laboratory animal by AWA, this study did not require Institutional Animal Care and Use Committee approval. The study was performed in accordance with the ethical rules set by Baylor College of Medicine.

Note that full information on the approval of the study protocol must also be provided in the manuscript.
